# Identification of New IκBα Complexes by an Iterative Experimental and Mathematical Modeling Approach

**DOI:** 10.1371/journal.pcbi.1003528

**Published:** 2014-03-27

**Authors:** Fabian Konrath, Johannes Witt, Thomas Sauter, Dagmar Kulms

**Affiliations:** 1 Institute of Cell Biology and Immunology, University of Stuttgart, Stuttgart, Germany; 2 Institute for System Dynamics, University of Stuttgart, Stuttgart, Germany; 3 Life Sciences Research Unit, University of Luxembourg, Luxembourg, Luxembourg; 4 Experimental Dermatology, Department of Dermatology, TU-Dresden, Dresden, Germany; Johns Hopkins University, United States of America

## Abstract

The transcription factor nuclear factor kappa-B (NFκB) is a key regulator of pro-inflammatory and pro-proliferative processes. Accordingly, uncontrolled NFκB activity may contribute to the development of severe diseases when the regulatory system is impaired. Since NFκB can be triggered by a huge variety of inflammatory, pro-and anti-apoptotic stimuli, its activation underlies a complex and tightly regulated signaling network that also includes multi-layered negative feedback mechanisms. Detailed understanding of this complex signaling network is mandatory to identify sensitive parameters that may serve as targets for therapeutic interventions. While many details about canonical and non-canonical NFκB activation have been investigated, less is known about cellular IκBα pools that may tune the cellular NFκB levels. IκBα has so far exclusively been described to exist in two different forms within the cell: stably bound to NFκB or, very transiently, as unbound protein. We created a detailed mathematical model to quantitatively capture and analyze the time-resolved network behavior. By iterative refinement with numerous biological experiments, we yielded a highly identifiable model with superior predictive power which led to the hypothesis of an NFκB-lacking IκBα complex that contains stabilizing IKK subunits. We provide evidence that other but canonical pathways exist that may affect the cellular IκBα status. This additional IκBα:IKKγ complex revealed may serve as storage for the inhibitor to antagonize undesired NFκB activation under physiological and pathophysiological conditions.

## Author Summary

In unstimulated cells, the transcription factor NFκB resides in the cytosol bound to its inhibitor IκBα. Canonical activation of NFκB by numerous stimuli leads to proteasomal depletion of IκBα, thereby liberating NFκB to translocate into the nucleus to induce transcription of genes leading to proliferation, angiogenesis, metastasis, or chronic inflammation. Consequently, only transient activity needs to be warranted by immediate NFκB-dependent induction of negative regulatory mechanisms, including up-regulation of its inhibitor IκBα. Resynthesized IκBα consequently terminates NFκB activity by binding to its nuclear localization sequence. However, under physiological or pathophysiological conditions, random NFκB activation may occur, which needs to be avoided in order to guarantee proper cellular function. Using detailed dynamical modeling, we have now identified an additional IκBα containing complex to exist in un-stimulated cells which lacks NFκB but includes IKKγ (IκBα:IKKγ complex). This additional IκBα is not depleted from cells in the canonical fashion and may therefore serve as a cellular backup to avoid random NFκB activation.

## Introduction

The nuclear transcription factor κB (NFκB) family consists of five DNA-binding proteins (p65, p50, p52, cRel, RelB) that differentially modulate gene transcription. Since NFκB activation is involved in many cellular processes including inflammation, proliferation, angiogenesis and anti-apoptosis, only transient expression of the responsive genes ensures proper function of living cells [1]. Impairment of the regulatory system may contribute to malignant transformation, invasion and metastasis [2]. Consequently, NFκB controls its own activity by initiating negative feedback mechanisms including transcriptional up-regulation of cellular inhibitors [3], [4]. Two independent pathways have been described to induce NFκB activation. While the non-canonical pathway determines the activity of p50:p52 heterodimers, the more prominent canonical pathway controls activation of p65:p50 subunits. In un-stimulated cells NFκB (p65:p50) resides inactively within the cytosol, bound to its inhibitor IκBα, which covers its nuclear localization signal [5]. Following canonical signal transduction, NFκB activation is triggered via the IκB kinase complex (IKK), which consists of two catalytic subunits, IKKα and IKKβ, as well as the regulatory subunit IKKγ. Site specific phosphorylation of IKKβ mediates downstream phosphorylation of IκBα at two Ser residues, serving as a signal for poly-ubiquitination and proteasomal degradation of the inhibitor. Liberated NFκB subsequently translocates into the nucleus to serve its function as a transcription factor which also – and most importantly – includes induction of negative feedback regulation via IκBα re-synthesis [6]. The network of interaction, however, leading to activation, inhibition or post-activational attenuation of NFκB is very complex and can be influenced by changes in signal transduction as well as through specific modifications of the molecules involved. Since dysregulation of NFκB plays a major role in the development of various diseases, a number of mathematical models have been implemented to analyze the non-linear dynamical behavior of this regulatory network [7], [8]. These models contributed to the analysis of the role of negative feedback loops [9]–[11], the description of the overall input-output behavior of the pathways [12]–[14], the understanding of the integration from multiple input signals [14], [15], as well as to the identification of sensitive parameters [16]. However, the pattern of NFκB-induced gene expression may significantly change depending on the cell type, the intracellular protein-protein interaction and the physiological context. Following this line, previous studies revealed canonical NFκB responses to dramatically change in cells being exposed to DNA-damaging agents, including ultraviolet-B (UVB) radiation. In particular, Interleukin-1 (IL-1) stimulation was shown to protect epithelial cells from death ligand-induced apoptosis via NFκB-dependent up-regulation of anti-apoptotic genes. When co-irradiated with UVB, instead, IL-1 driven and NFκB-dependent repression of anti-apoptotic genes caused enhancement of UVB-induced apoptosis [17]. As a prerequisite, nuclear persistence of NFκB was shown to be facilitated via UVB-induced inactivation of the catalytical subunit of Ser/Thr phosphatase PP2A, causing chronic IKKβ activation and subsequent phosphorylation and proteasomal degradation of resynthesized IκBα. Both modifications in concert were shown to drive the pro-apoptotic properties of a classical non-apoptotic protein [3], [18]. Using a systems biological approach we could show that not only PP2A inactivation but also global translational inhibition appeared to be involved in preventing IκBα recurrence. Above this, translational inhibition was shown to induce IκBα depletion in cells irradiated with UVB alone, indicating both mechanisms to individually mediate UVB-dependent responses [15], [19].

Similar to UVB, co-stimulation of cells with IL-1 and the tyrosine phosphatase inhibitor orthovanadate (OVA) induced tyrosine kinase cSrc-mediated inactivation of PP2A. Again here, chronic phosphorylation of IKKβ and consequently inhibition of IκBα recurrence provided for sustained NFκB activation [18]. In the present study we aimed to understand which additional regulatory mechanisms may exist to prevent unwanted NFκB activation under physiological conditions. We therefore analyzed OVA-dependent IκBα depletion as a tool to identify additional IκBα sources within the cell by iterative model refinement in combination with model inspired experimentation. The extended model predicted alternative non-canonical IκBα-degradation to occur without affecting NFκB activity. Respective experimental design finally revealed a yet unknown IκBα:IKKγ complex to exist, which might serve as a backup for negative feedback regulation of NFκB.

## Results

### OVA treatment alone causes incomplete IκBα depletion

**Figure 1:**
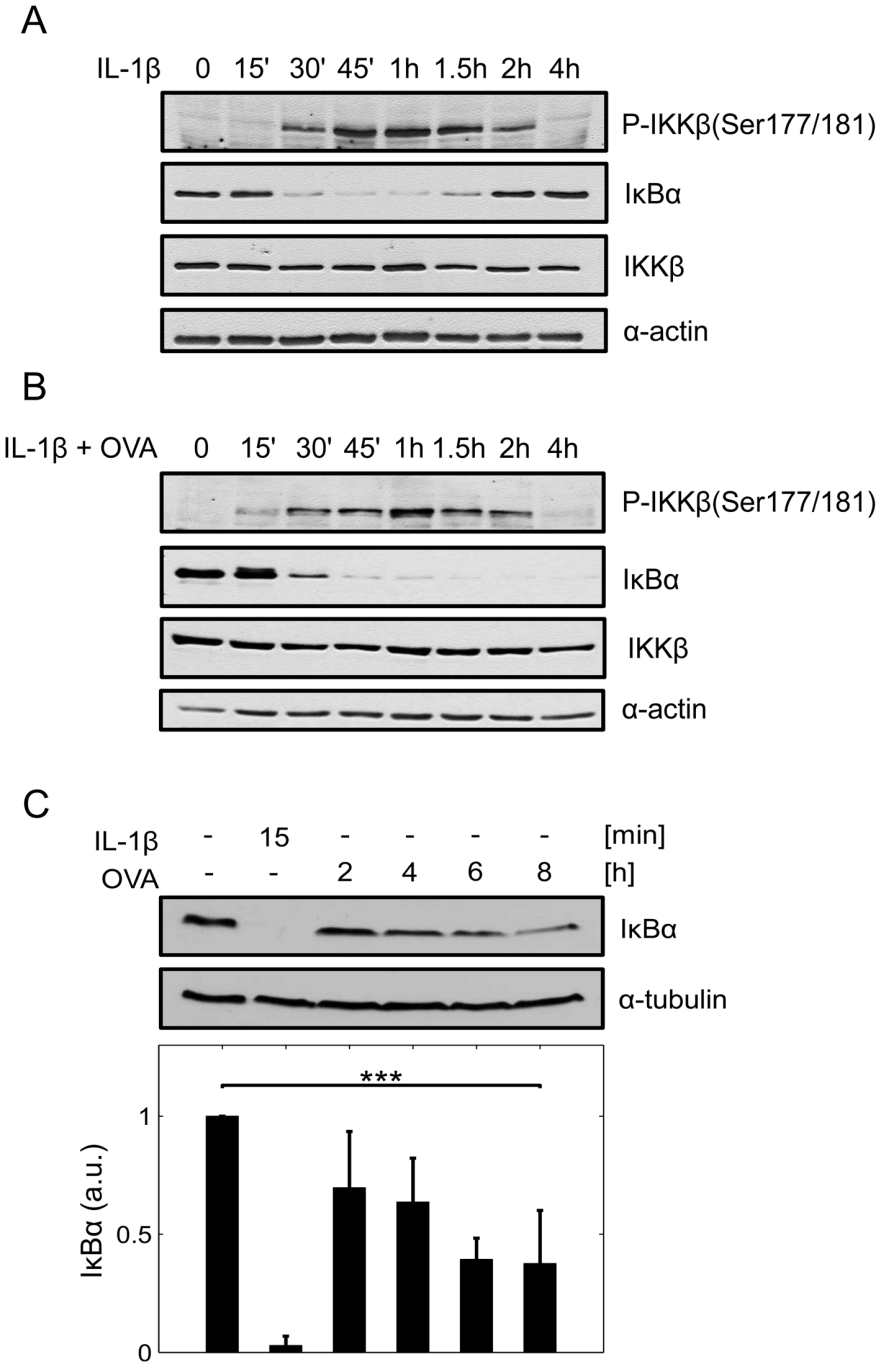
OVA promotes delayed depletion of IκBα independent of IKKβ phosphorylation. **A:** KB cells were left untreated or pre-treated with OVA (1 mM) for 1 h followed by stimulation with IL-1 (10 ng/ml). At the indicated time points following IL-1 stimulation the cellular status of P-IKKβ (Ser177/181), IκBα and IKKβ was determined by Western-blot analysis. Equal loading was monitored with an anti-α-actin antibody. **B:** Cells were left untreated, stimulated with IL-1 or OVA for the indicated time points and the cellular IκBα status determined by Western-blot analysis, α-tubulin served as a loading control, ***p<0.001.

Stimulation of cells from the epithelial cell line KB with IL-1 caused canonical degradation of the NFκB inhibitor IκBα via phosphorylation of the upstream kinase IKKβ at Ser177/181. Perfectly in line with the phosphorylation pattern of IKKβ, IκBα was completely degraded 30 min after IL-1 stimulation and became resynthesized after 2 h when phosphorylation of IKKβ started to descent (Fig. 1A). Co-treatment of cells with IL-1+OVA instead accelerated phosphorylation of IKKβ causing early phosphorylation and degradation of IκBα (Fig. 1B, 15 min). Strikingly, re-accumulation of IκBα was completely inhibited under these conditions even though the phosphorylation pattern of IKKβ at later time points (2 h; 4 h) remained largely unchanged compared to IL-1 treated cells (Fig. 1A). This strongly indicated that other OVA-driven mechanisms may superimpose IL-1-mediated canonical IκBα degradation at later time points. In accordance with this assumption we could detect partial, almost linear IκBα depletion at 4–8 h after OVA only treatment, following a much slower kinetics than canonical IL-1-driven IκBα degradation (Fig. 1C). Since IKKβ phosphorylation did not seem to play a major role in delayed OVA-dependent IκBα depletion we next examined whether transcriptional or translational inhibition might be involved.

### IκBα depletion is independent of transcriptional and/or translational inhibition

**Figure 2:**
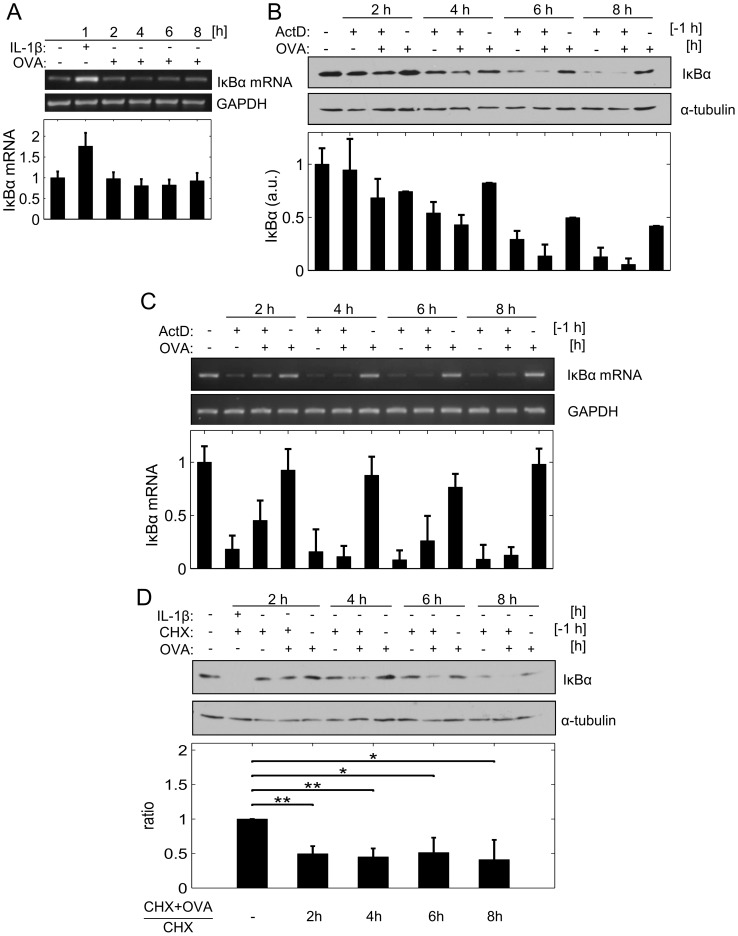
OVA-dependent IκBα depletion is mediated at the post-translational but not at the transcriptional level. **A:** Cells were left untreated, stimulated with IL-1 (10 ng/ml) or OVA (1 mM). At the indicated time points transcriptional regulation of IκBα was monitored by RT-PCR, with GAPDH serving as a loading control. **B:** Cells were pre-treated or not with ActD (5 µg/ml) for 1 h and subsequently stimulated or not with OVA (1 mM) as indicated. Protein level of IκBα was determined by Western-blot analysis with α-tubulin as loading control, and **C:** transcription level of IκBα by RT-PCR with GAPDH as loading control. **D:** Cells were pre-treated or not with CHX (5 µg/ml) for 1 h and subsequently stimulated or not with OVA (1 mM) as indicated. Protein level of IκBα was determined by Western-blot analysis with α-tubulin serving as a loading control, **p<0.005; *p<0.05.

Performing RT-PCR analysis we revealed the IκBα mRNA level to remain completely unchanged, even up to 8 h after OVA treatment, while being up-regulated 1 h after canonical IL-1 treatment, as a positive control (Fig. 2A). Application of the transcription inhibitor actinomycin D (ActD) strongly implied transcriptional alterations not to be involved in OVA-induced IκBα depletion. While OVA treatment alone induced a moderate and incomplete reduction of the IκBα protein (Fig. 2B) but not the respective mRNA (Fig. 2C) over time, transcriptional inhibition by ActD caused pronounced inhibition of both the mRNA and the protein level of IκBα, respectively. Of note, co-application of OVA and ActD further enhanced depletion of IκBα protein without additively affecting the transcription level (compare Fig. 2B + 2C). Results indicated that OVA-induced IκBα depletion is facilitated at the protein level, independent of transcriptional regulation. An analogous IκBα protein pattern was obtained when inhibiting translation by addition of cycloheximide (CHX). While individual treatment with either CHX or OVA caused IκBα reduction over time, co-application of both substances additively enhanced loss of IκBα (Fig. 2D). These data strongly support the concept that OVA-mediated IκBα depletion is independent of translational inhibition but might be caused by activation of upstream signaling pathways apart from canonical NFκB signal transduction.

### OVA-induced IκBα depletion neither follows the canonical pattern nor causes NFκB activation

**Figure 3:**
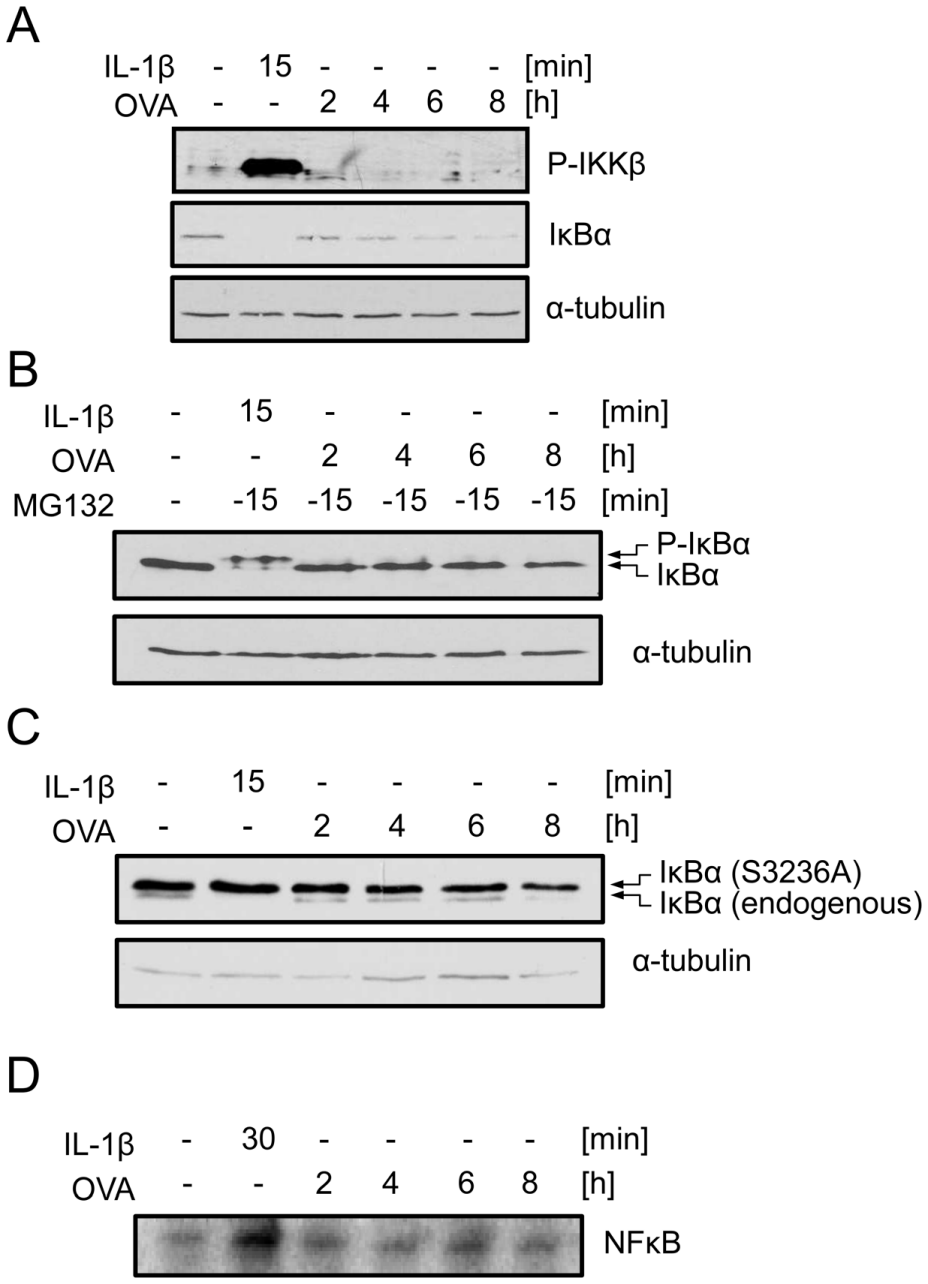
OVA-induced IκBα depletion does not follow the canonical NFκB activation pattern. **A:** Cells were stimulated with IL-1 (10 ng/ml) or OVA (1 mM) for the indicated time points. Status of p-IKKβ and IκBα was determined by Western-blot analysis. **B:** Cells were pretreated with the proteasome inhibitor MG132 for 15 min. Subsequently cells were stimulated as in **A** and the IκBα status determined by Western-blot analysis. **C:** KB cells stably expressing the IκBα super-repressor variant Ser32/36Ala were stimulated as cells in **A**, and the IκBα status determined by Western-blot analysis. **D:** Cells were stimulated as in **A**, nuclear protein extracts were isolated and subjected to EMSA using an NFκB-specific consensus sequence.

Canonical NFκB activation is well known to involve Ser177/181 phosphorylation of IKKβ, followed by Ser32/36 phosphorylation of IκBα as a prerequisite for its proteasomal degradation. Stepwise documentation of canonical NFκB activation by Western-blot analysis and electro mobility shift assay (EMSA) revealed that OVA-induced IκBα depletion does not follow the canonical pattern. While IL-1-induced total IκBα degradation occurred as a fast process being completed after 15 min, OVA-induced subtotal IκBα depletion followed a much slower kinetics, and did not involve classical IKKβ phosphorylation (Fig. 3A). Illustrating canonical IκBα degradation by addition of the proteasome inhibitor MG132, which stabilizes phosphorylated IκBα, revealed only canonical IL-1 stimulation to cause an IκBα shift, while OVA treatment did not, but still caused IκBα depletion over time (Fig. 3B). Correspondingly, OVA depleted not only IκBα-*wt* but also the Ser32/36Ala mutant which lacks the IKKβ-dependent phosphorylation sites and can therefore not be degraded in the canonical fashion (Fig. 3C). Interestingly, EMSA revealed no significant nuclear translocation of NFκB to occur upon delayed OVA-induced IκBα depletion (Fig. 3D), indicating other than NFκB-bound IκBα pools to exist within un-stimulated cells. In order to identify this additional IκBα pool we integrated all experimental data into detailed dynamical modeling using ordinary differential equations.

### IκBα co-exists within different protein complexes

**Figure 4:**
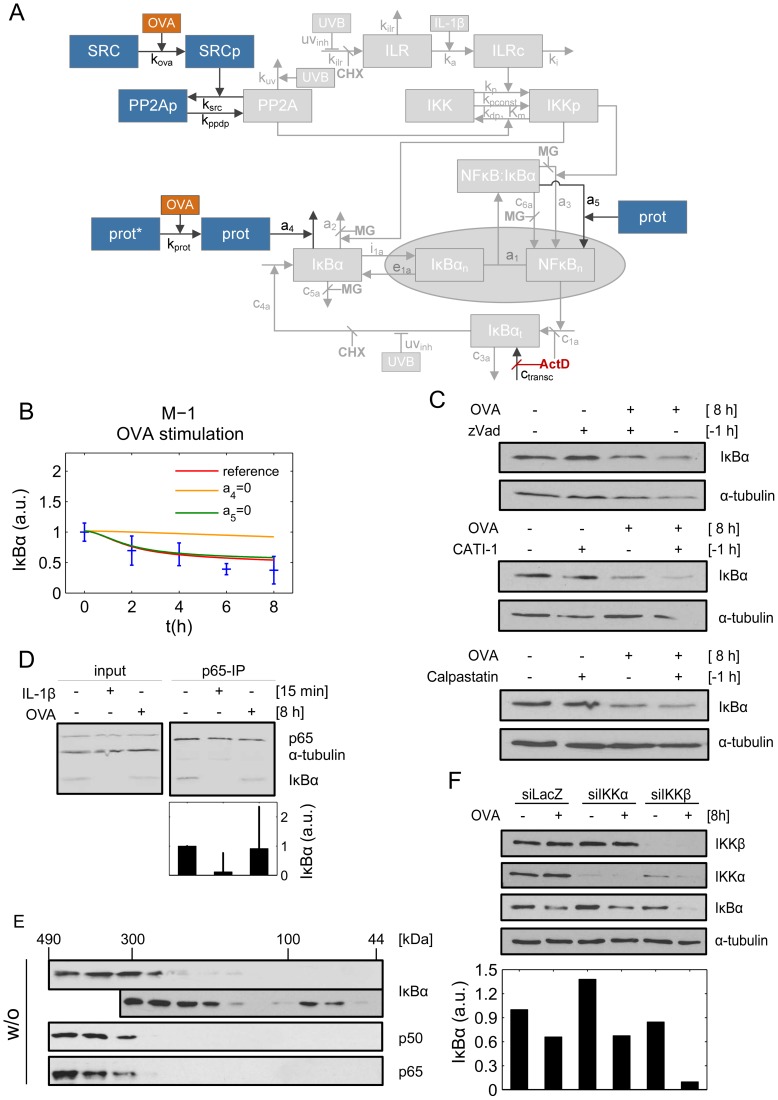
IκBα co-exists in different cellular complexes. **A:** Schematic representation of model variant M-1. Input (IL-1, UVB, OVA, MG132 (MG), ActD, CHX) is given in orange and red, respectively. Reactions and variables adopted from our previous model (Witt et al, [15]) are shown in grey. **B:** The red line represents simulation data of the best fit (reference). The impact of protease-mediated degradation of free IκBα is depicted in orange and determined by setting the rate constant of this reaction *(a_4_*) to zero. Likewise, the rate constant of protease-mediated degradation of NFκB bound IκBα (*a_5_*) was set to zero (green line). Experimental data and standard deviation is shown in blue. **C:** KB cells were left untreated, pre-stimulated with zVAD (20 µM), CATI-1 (50 µM) or calpastatin (1 µM), for 1 h followed by OVA treatment for 8 h. Protein level of IκBα was determined by Western-blot analysis with α-tubulin serving as a loading control. **D:** Cells were left untreated, stimulated with IL-1 for 15 min or OVA for 8 h. Subsequently protein extracts were subjected to immunoprecipitation with an antibody specific for the p65 subunit of NFκB. The amount of co-precipitated IκBα was determined by Western-blot analysis compared to input protein levels. Bars represent IκBα levels that are normalized to the respective p65 levels. **E:** Lysates from unstimulated cells were subjected to size exclusion chromatography. Individual fractions were analyzed via Western-blotting, using antibodies against IκBα, p65 and p50. **F:** IKKα and IKKβ, respectively, were transiently knocked down using siRNA, with siLacZ serving as a negative control. Subsequently cells were stimulated with OVA for 8 h and the IκBα status, as well as IKKα and IKKβ knock down determined by Western-blot analysis. α-tubulin served as loading control.

Based on our previous NFκB signaling model [15] we generated an extended mathematical model (variant M-1, Fig. 4A). Since OVA-mediated IκBα depletion appeared to be independent of both, transcriptional/translational inhibition and proteasomal degradation, we assumed proteases to be involved in this process. It is known that free as well as NFκB-bound IκBα can be degraded by proteases in a proteasome- as well as in a lysosome-independent manner [20], [21]. To monitor the role of OVA within the entire NFκB signaling network, we also included OVA-dependent Src phosphorylation - which subsequently leads to inactivation of PP2A and chronic IKKβ activation - into the model. This part of the signaling pathway, however, exclusively plays a role at early time points during IL-1 dependent canonical IκBα degradation [18]. Successful parameter fitting was performed based on a huge variety of experimental data comprising multiple stimulations: OVA−, ActD−, ActD+OVA, CHX+OVA-stimulation (Fig. 1–3), IL-1 + OVA-stimulation [18], as well as data describing IκBα depletion upon IL-1−, UVB−, IL-1+UVB−, CHX−, IL-1+UVB+MG132-stimulation collected before [15], [19]. All experimental data could be fitted well (Fig. S1). In particular, the good reproducibility of OVA experiments indicated that addition of an IκBα-degrading protease is sufficient to reproduce OVA-induced IκBα depletion without activating NFκB. The model thereby predicted a pool of free IκBα which is eliminated by a putatively OVA-activated protease, but leaves NFκB-bound IκBα almost unaffected. In the best fit scenario of knock down simulation studies depletion of free IκBα showed major relevance while the depletion of NFκB bound IκBα is negligible (Fig. 4B). Of note, OVA-induced PP2A inactivation is only important in IL-1 stimulated cells and is therefore also irrelevant for OVA only stimulation following a much slower kinetics.

Still, the model predictions had to deal with two obstacles: Firstly, due to its immense instability only a very small pool of free IκBα seems to exist within the cell at all [22]. Secondly, proteolytic cleavage of IκBα at least by three major groups of proteases: caspases, calpains and cathepsins could be excluded by the use of specific inhibitors (Fig. 4C). To follow up the model based hypothesis of a stably existing IκBα form that is not bound to NFκB, we immuno-precipitated the NFκB p65 subunit from whole cell lysates and checked the levels of co-precipitated IκBα in un-stimulated *versus* OVA-treated cells. The amount of IκBα remained largely unchanged, whereas IκBα was absent in cells stimulated with IL-1, due to complete canonical degradation (Fig. 4D). These data strongly supported the assumption that only IκBα which is not bound to NFκB is depleted in an OVA-dependent fashion. To investigate whether depleted IκBα in fact is free or bound to other cellular components but NFκB, we conducted size exclusion chromatography. Indeed, IκBα appeared to exist in at least three different forms in un-stimulated cells. While only a minimal fraction seemed to refer to unbound IκBα eluting at a size of 44 kDa, surprisingly no complex exclusively consisting of IκBα and NFκB (p65:p50) seemed to exist. Instead, complexes of higher molecular weight containing IκBα, p65 and p50 (NFκB) showed up at sizes ranging between 300 kDa and 490 kDa, indicating to incorporate other proteins as well. In addition, different aggregates ranging between 100 kDa and 300 kDa in size appeared which did not contain any NFκB components, implying IκBα to also form complexes with other proteins (Fig. 4E). To investigate whether IKKs might be involved in stabilizing IκBα we knocked down the two catalytic subunits IKKα and IKKβ by RNA interference. While IKKα knock down had no effect on IκBα depletion, knock down of IKKβ seemed to enhance loss of IκBα, implying that binding to IKKβ stabilizes IκBα which is not bound to NFκB (Fig. 4F). The fact that model variant M-1, and a simple extension by adding a second (competing) IκBα complex formation (variant M-2, Fig. S3), respectively, could not fully explain the IKK knock down data (Fig. S2 and S4) argues for an additional IκBα-IKK interaction and calls for a more in depth analysis of the IκBα complexes.

### Novel cellular IκBα complexes containing IKK components but no NFκB

**Figure 5:**
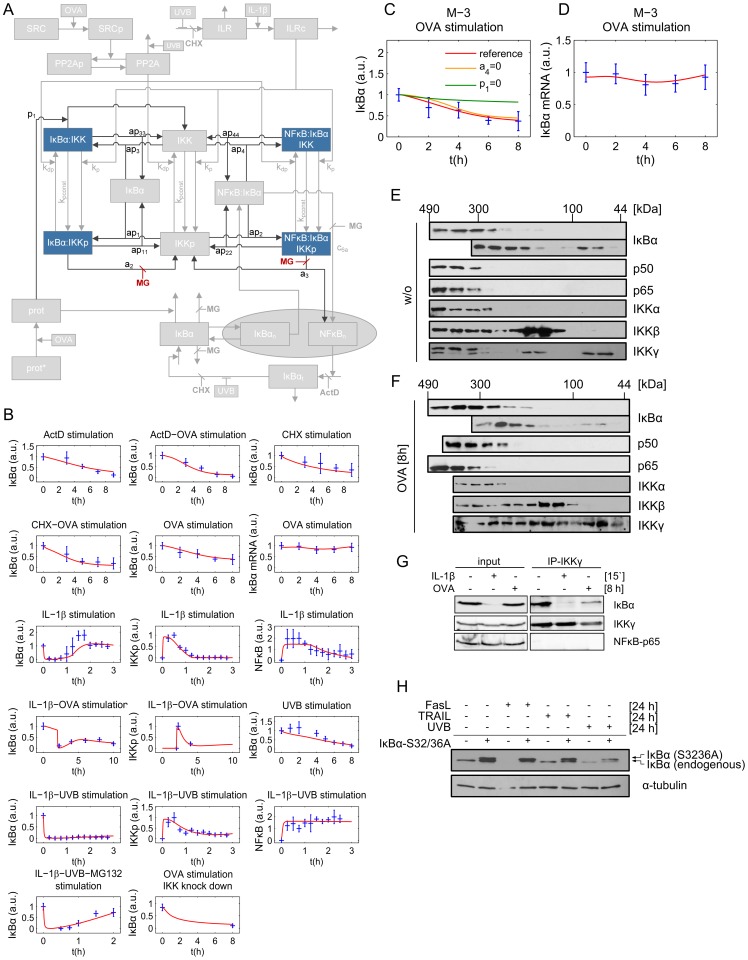
NFκB free IκBα complexes contain subunits of the IKK complex. **A:** Schematic representation of model M-3 (final model). State variables are depicted in blue, inputs (IL-1, UVB, OVA, MG132 (MG), ActD, CHX) in orange and red, respectively. Reactions and variables adopted from our previous model (Witt et al, [15]) or model variant M-1 or M-2 are shown in grey. **B:** Overall simulation results of the best fit. The 127 data points and standard deviations are depicted in blue, the red line represents simulated time course of the respective state variable. The overall χ^2^ value is 100.6. **C:** Reference (red line) represents simulation data of the best fit. Setting the rate constant of protease-mediated degradation of free IκBα (*a_4_*) to zero reveals the impact of this reaction on observed IκBα degradation (orange). The impact of protease-mediated degradation of IKK bound IκBα (*p_1_*) is depicted in green. **D:** Simulated time course of the IκBα mRNA level after 8 h of OVA treatment is depicted in red. Corresponding experimental data and standard deviation is shown in blue. Lysates from **E:** unstimulated or **F:** OVA treated cells were subjected to size exclusion chromatography. Individual fractions were analyzed by Western-blotting, using antibodies against IκBα, p65 and p50, IKKα, IKKβ and IKKγ. **G:** Cells were left untreated, stimulated with IL-1 for 15 min or OVA for 8 h. Subsequently IKKγ was immunoprecipitated from protein extracts and the amount of co-precipitated NFκB (p65) and IκBα determined by Western-blot analysis compared to input protein levels. **H:** KB cells stably expressing the IκBα super-repressor variant Ser32/36Ala were stimulated as indicated. After 24 h cellular IκBα status was monitored by Western-blot analysis with α-tubulin serving as a loading control.

To stress whether IKK components play a role in IκBα complex formation we first generated model variant M-3 assuming that IKK is permanently bound to IκBα which lacks NFκB (Fig. 5A). According to the chosen model structure, binding of IKK to IκBα results in the formation of an IκBα:IKK as well as an NFκB:IκBα:IKK complex. As phosphorylated IKK (IKKp) initiates degradation of IκBα, the IKKp containing complexes are highly unstable and dissociate into free IKKp and free NFκB which translocates into the nucleus.

The simulation results of this model variant revealed a very good reproducibility of all experimental data including the OVA+IKK knock down experiment (Fig. 5B).

When exploring the model predicted steady state levels in the un-stimulated IKK knock down setting (Fig. S5), very low IKK yielded decreased formation of both IKK containing complexes IκBα:IKK and NFκB:IκBα:IKK. As a consequence elevated levels of free and NFκB-bound IκBα (NFκB:IκBα) were predicted. Consequently, constitutive degradation of NFκB:IκBα results in enhanced NFκB activation and further increased levels of free IκBα (Fig. S5A). Starting from these changed steady state levels in the IKK knock down setting - especially taking the increased level of free IκBα into account - the OVA-mediated degradation of IκBα could be reproduced accurately (Fig. S6).

In this final model OVA mediated the degradation of an NFκB-free IκBα complex (IκBα:IKK), thus preventing activation of NFκB and IκBα mRNA synthesis (Fig. 5C and Fig. 5D).

In summary, the mathematical model based analysis clearly proposes the existence of IKK containing IκBα complexes lacking NFκB, supporting the hypothesis that IKKβ stabilizes “unbound” IκBα. Size exclusion chromatography clearly revealed that the high molecular weight complexes (300–490 kDa) in un-stimulated cells consisted of at least IκBα, NFκB (p65:p50), and all three IKK components IKKα, IKKβ and IKKγ. Most importantly, IKKγ eluted together with IκBα at 100 kDa, perfectly matching the size of a heterodimeric complex (±94 kDa). Of note, additional complexes containing exclusively IKKβ and IKKγ (±140 kDa) but not IKKα appeared to be formed (Fig. 5E).

Treatment with OVA for 8 h did not significantly change the composition of the high molecular weight complexes. In contrast, it caused pronounced depletion of IκBα from the IκBα:IKKγ complex, while IKKγ seems to randomly distribute over numerous fractions. This clearly indicates dissociation of this heterodimeric IκBα:IKKγ complex to precede IκBα depletion (Fig. 5F). The existence of an NFκB-lacking IκBα:IKKγ complex as well as the specific depletion of IκBα from this particular complex following OVA treatment was confirmed by co-immunoprecipitation. While in unstimulated cells reasonable amounts of IκBα but no NFκB were shown to bind to IKKγ, the level of IκBα decreased significantly upon treatment of cells with OVA for 8 h. No IκBα was found bound to IKKγ in IL-1 stimulated cells, due to complete canonical degradation (Fig. 5G). This is perfectly in line with simulation results derived from the final model variant M-3 suggesting that degradation of NFκB-free but IKK-bound IκBα is responsible for the partial IκBα depletion in response to OVA.

To assess the functional relevance under physiological conditions, we investigated IκBα depletion in response to stimuli being capable to induce both, NFκB activation and apoptotic cell death [23], [24]. Treatment with the death ligands TRAIL and FasL, respectively, exclusively caused canonical IκBα degradation, being indicative by sparing the IκBα super-repressor variant from depletion. In contrast, irradiation of cells with UVB additionally resulted in IκBα depletion independent of the canonical pathway – represented by degradation of both, the endogenous and the super-repressor variant (Fig. 5H). These data strongly support the notion that other but canonical pathways exist that may affect the status of IκBα within the cell. Thus, we have uncovered an additional IκBα complex to exist in un-stimulated cells that might serve as storage to antagonize random or undesired NFκB activation and consequently ensures proper cellular function.

## Discussion

In the past decades activation of NFκB has exclusively been attributed to either canonical or non-canonical signal transduction pathways. Both pathways are tightly regulated by a series of phosphorylation and ubiquitination events that cause nuclear translocation of distinct NFκB family members. Canonical signal transduction basically accumulates at two well described protein complexes, namely IKK and NFκB:IκBα. Accordingly, stimulation of cells with the pro-inflammatory cytokine IL-1 causes downstream activation of the IKK complex, in particular the catalytic subunit IKKβ, to mark IκBα for proteasomal degradation. Released NFκB in turn triggers resynthesis of its inhibitor in a negative regulatory feedback loop. Most recently, a number of ubiquitin ligases including TRAF molecules and the lubac complex have additionally been implemented in canonical NFκB activation [25], [26], while de-ubiquitinases like A20 and CYLD serve a well-known function in negative feedback regulation [27], [28]. In the present study we provide evidence that besides the well-known components of canonical NFκB signaling, alternative IκBα containing complexes exist within the cell that might indirectly contribute to NFκB regulation. Short term inhibition of IκBα resynthesis following IL-1+UVB and IL-1+OVA treatment, respectively, is due to abrogation of canonical negative feedback regulation via inhibition of PP2A leading to chronic IKKβ activation [3], [19]. At later time points, however, canonical feedback regulation seems to be superimposed by an IKKβ-independent OVA- driven mechanism that follows a slower kinetics and only causes partial IκBα depletion. A similar observation could previously be made in UVB-irradiated cells, showing slow and subtotal IκBα depletion resulting in only moderate and delayed NFκB activation [3]. In the present study OVA-induced delayed IκBα depletion appeared to follow a pattern different from canonical NFκB activation, because super-repressor variants of IκBα could be depleted as well. This implies IκBα depletion which does not follow the canonical pattern to serve an important, yet unknown function. Although a number of alternative ways to proteolytically cleave IκBα have been described in the literature [20], [21], [29], [30] inhibition of the major cellular protease families, could be ruled out. More recently an alternative proteasome independent mechanism called PIR has been described to enhance IκBα turnover in B-cells, however, PIR resulted in constitutive p50:cRel activation in those cells and may therefore play a different role [21], [31]. Still, integration of an OVA-activated protease into all our mathematical models that is able to deplete IκBα- different from the canonical mechanism - was shown to nicely reproduce the OVA induced IκBα degradation with slow activation kinetics determined by a small rate constant (M-3: *k_prot_* = 3.76e^−7^ s^−1^, Table S1). Accordingly, significant degradation of IκBα occurs at later time points (4 h–8 h) and may involve PIR-like mechanisms.

Reproducing OVA-induced IκBα depletion without NFκB activation in model variant M-1 predicted high levels, 31%, of free IκBα (0.041 µM out of 0.135 µM) to exist within the cell (Table S2), whereas only 10% to 15% of free IκBα is supposed to exist [32], [33]. Remarkably, inclusion of putative NFκB-lacking IκBα complexes nicely scaled down the level of free IκBα to 13% (M-2) and 8% (M-3) respectively which now is in very good accordance to the literature values, matches mathematical models of other groups [11], [22], [34], and could also be verified by size exclusion chromatography.

According to previous studies that revealed IKKα and IKKβ to form high molecular complexes that contain IκBα as well as NFκB components [35], [36] model variant M-3 predicted IκBα to form both, stable IκBα:IKK as well as NFκB:IκBα:IKK complexes. With this final model the entire data of numerous experiments could be reproduced with a good fit quality. Finally formation of an IκBα:IKKγ complex could be verified experimentally by gel filtration analysis as well as co-immunoprecipitation. Expanding the scope of our previous model [15] by iterative model refinement revealed new insights into NFκB regulation and allowed to analyze the effect of an *in silico* knock down of the IKK complex on the OVA-mediated IκBα depletion. The model predicts that knocking down IKKβ enhances the level of an IκBα compound that can be degraded by the proposed protease. *In vitro* neither IκBα from the high molecular complex nor IKKβ:IKKγ bound IκBα seems to be depleted after OVA treatment. Thus knocking down IKKβ could result in decreased levels of NFκB:IκBα:IKK as well as IκBα:IKKβ:IKKγ and an elevated concentration of the IκBα:IKKγ complex. Due to larger amounts of IκBα:IKKγ, OVA-mediated degradation of this IκBα component would explain why IKKβ knock down enhances OVA-induced IκBα depletion. Additionally, a constant basic IKKβ-mediated IκBα turnover exists in unstimulated cells [19], yielding in continuous NFκB-dependent resynthesis of IκBα. In case of IKKβ knock down, this process is completely abrogated consequently causing subtle loss of IκBα over time exclusively dependent on the individual half life in treated versus untreated cells (see also Fig. 4F).

Combining experimental methods and detailed dynamical modeling we could provide strong evidence for the existence of an NFκB-free, IKKγ containing IκBα complex presumably acting as a cellular backup pool to capture randomly released NFκB. Above this, our model introduces an IκBα degradation pathway that is independent from canonical processes but is likely to influence the cellular status of IκBα. Our final mathematical model (M-3) created here is able to reproduce a huge number of datasets comprising various intracellular proteins and a wide range of stimulation experiments to extensively reflect on the whole NFκB signaling network above individual top-down signaling pathways. Thus, our model can be used for further modeling approaches regarding NFκB regulation and may provide predictive potential for sensitive parameters that may serve as therapeutic targets in the future.

## Materials and Methods

### Cells and reagents

The human epithelial carcinoma cell line KB (ATCC) was cultured in RPMI 1640, 10% FCS. Recombinant human IL-1β (R&D Systems, Wiesbaden, Germany) was applied at 10 ng/ml and Na-Orthovanadate (Sigma, Munich, Germany) at 1 mM. Actinomycin D and cycloheximide (Sigma) were added to cells at 5 µg/ml, respectively. Specific protease inhibitors (Calbiochem, Darmstadt, Germany) were applied at 50 µM for the cathepsin inhibitor CATI-1, 1 µM for calpastatin and 20 µM for the pan caspase inhibitor zVAD. Proteasomal inhibition was achieved by addition of 25 µM MG132 (Calbiochem). For Fas/CD95 receptor activation 0.5 µg/ml of an agonistic antibody (Immunotech, Monrovia, CA, USA) was used. Recombinant human iz-TRAIL protein, N-terminally fused to an isoleucine-zipper motif in order to constitutively build the trimerized active form [37] was kindly provided by Dr. Henning Walczak, Centre for Cell Death, Cancer and Inflammation, UCL, London and added at 100 ng/ml. UVB irradiation (300 J/m^2^) was performed with TL12 fluorescent bulbs (290–320 nm, Philips).

### Immunoprecipitation and WB analysis

Cells were lysed in lysis buffer (50 mM Hepes, pH 7.5; 150 mM NaCl; 10% glycerol; 1% Triton-X-100; 1.5 mM MgCl_2_; 1 mM EGTA; 100 mM NaF; 10 mM pyrophosphate, 0.01% NaN_3_ and Complete protease inhibitor cocktail; Roche, Mannheim, Germany) for 20 min on ice. Endogenous NFκB (p65) or IKKγ were immune-precipitated using specific antibodies (sc-372; sc-8330 Santa Cruz, Heidelberg, Germany) and A/G-plus agarose (Santa Cruz) over night. Precipitates were analyzed by Western-blotting using antibodies against NFκB (F6, sc-8008, Santa Cruz), IκBα (L35A5, Cell Signalling, Beverley, MA, USA) and IKKγ (IMG-324A, Imgenex, San Diego, CA, USA). For WB analysis cells were lysed by addition of hot (95°C) Laemmli buffer. 80 µg protein extracts were subjected to SDS-PAGE and Western-blot analyses using antibodies against IκBα, P-IKKβ-Ser177/181, IKKβ, p50 (L35A5, 16A6, 2C8, #3035, Cell Signaling), p65 (sc-8008, Santa Cruz), IKKα (556532, BD Biosciences), IKKγ (IMG-324A Imgenex, San Diego, CA, USA), and α-tubulin (DM1A, Neomarkers, Fremont, CA, USA), using West-Pico or West-Dura (Pierce, Thermo Scientific, Rockford, IL, USA) chemiluminescent substrates.

### Semiquantitative RT-PCR analysis

Total RNA was extracted from cells using GIT-buffer (4 M guanidinthiocyanate, pH 4.8; 0.3 M NaOAc; 1% N-lauroylsarcosine; 0.2% β-mercaptoethanol) followed by phenol/chloroform extraction utilizing Phase Lock Heavy tubes (Eppendorf AG, Hamburg, Germany). Six µg of total RNA was reverse transcribed with an AMV Reverse Transcriptase kit (Promega, Mannheim, Germany). The following primers were used in a 20 µl reaction utilizing the RedTaq polymerase system (Sigma):

GAPDH:

F: 5′-GCCTCCTGCACCACCAACTGC-3′; R: 5′-CCCTCCGACGCCTGCTTCAC-3′

IκBα:

F: 5′-ACAGGAATTACAGGGTGCAGG-3′; R: 5′-GAGAAACTCCCTGCGATGAG-3′

### Plasmids and transfection of cells

For ectopic expression of IκBα -S32/36A 6,5×10^6^ cells were transfected with 25 µg of the respective pcDNA3.1-based construct by electroporation at 1200 µF and 250 V (EasyjecT-plus, Peqlab, Erlangen, Germany) in ice cold RPMI medium w/o FCS. Transfection efficacy ranged between 70 and 80%.

### RNA interference

1×10^5^ cells were transfected with 0.5 pmol/µl siRNA knocking down IKKα: GCAGAAGAUUAUUGAUCUATT or IKKβ: UUCAGAGCUUCGAGAAGAATT (Eurofins MWG, Ebersberg, Germany) using Lipofectamin 2000 (Life Technologies, Darmstadt, Germany) according to the protocol. Cells were analyzed after 72 h.

### Electro mobility shift assay (EMSA)

Following stimulation cells were harvested and nuclear proteins extracted as described before [38]. The NFκB consensus oligo nucleotide (sc-2505; Santa Cruz) was end-labeled using [γ^32^P] ATP and T4 polynucleotide kinase (MBI Fermentas, Ontario, Canada), followed by column-purification (QIAquick Nucleotide Removal Kit, Qiagen, Hilden, Germany). Binding reactions were carried out in a 20 µl volume containing 8 µg nuclear protein extract in 5× binding buffer (20 mM HEPES, pH 7.5; 50 mM KCl; 2.5 mM MgCl_2_; 20% (w/v) ficoll; 1 mM DTT), containing 1 µg poly[dIdC]; 2 µg BSA, and 70.000 cpm of ^32^P-labeled NFκB consensus oligo nucleotide for 20 min at RT. Samples were separated on a 4% native PAGE at 150 V for 2.5 h and detected by autoradiography.

### Size exclusion chromatography (FPLC)

Cells were harvested in PBS+0.01% sodium acide and disrupted by sonication. 10 mg protein extract was fractionated on an agarose bead column (GE, Healthcare, Frankfurt, Germany). Fractionated proteins were precipitated in 50% TCA, washed twice with acetone and applied to SDS-PAGE and subsequent Western-Blot analysis. Proteins of known size (thyroglobulin: 670 kDa, γ-globulin: 158 kDa, ovalbumin: 44 kDa, myoglobin: 17 kDa, cobalamin: 1.3 kDa) were used as a standard.

### Data processing

Western-Blots analyses are presented as mean ± SD of 3 independently performed experiments. Blots were evaluated densitometrically and the results normalized to the maximal measured value. A minimal relative standard deviation of 15% was always assumed. For statistical analysis student's t-test was performed.

### Mathematical modelling

The mathematical models created in this study are based on our previous ODE model [15] comprising the following components:

IL-1-receptor (IL-1R), IL-1-receptor ligand complex (ILRc), free IκBα (IκBα), NFκB bound IκBα (NFκB:IκBα), nuclear IκBα (IκBα_n_), nuclear NFκB (NFκB_n_), IκBα mRNA (IκBα_t_), IKKβ (IKK), phosphorylated IKKβ (IKKp), Protein phosphatase 2A (PP2A).

In our recent models IKK (and IKKp) is perceived as the IKK complex consisting of IKKα, IKKβ and IKKγ instead of IKKβ alone.

First of all we extended the basic model [15] by our previously proposed mechanism of OVA prolonging the IL-1 mediated activity of NFκB [18]. OVA treatment causes phosphorylation of the tyrosine kinase cSrc which in turn phosphorylates and thereby inactivates PP2A:
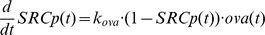


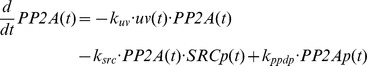


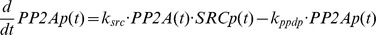



Terms adopted from the model of Witt et al. [15] are highlighted in bold font. PP2Ap represents phosphorylated PP2A. Without loss of generality the total amount of the kinase cSrc (SRC) can be assumed to be 1 (cf. an analogous reasoning for IKK in [19]). Considering mass conservation, (*1-SRCp(t)*) then represents the Src kinase in its inactive (un-phosphorylated) state. The stimulus OVA is represented by the step function *ova(t)* having a value of 0 (absent) or 1 (present). The initial concentration of PP2A is set to 1 whereas PP2Ap is set to zero. In order to reach steady state conditions we started stimulation from steady state established after 240 hours of simulation of the un-stimulated system.

Based on our recent results we assumed for the observed OVA mediated IκBα degradation the involvement of a protease *prot* that is activated by OVA. This assumption is included in all of our model variants by adding the following term:
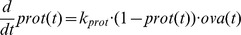



In this equation *prot* represents the active form of the assumed protease and *(1-prot(t))* the inactive state considering mass conservation. The total amount of *prot* is assumed to be 1 without loss of generality. In model variant M-1 this protease is able to degrade free and NFκB bound IκBα:
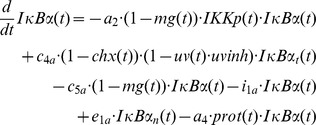


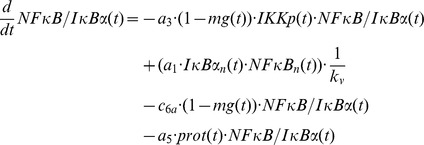



Degradation of NFκB bound IκBα by the protease in model variant M-1 causes translocation of NFκB into the nucleus followed by the initiation of IκBα mRNA synthesis:
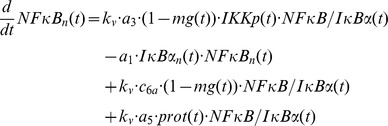


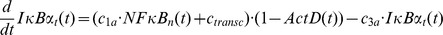



The factor *k_v_* is used to compensate the different volumes of cytosol and nucleus (see Witt et al. [15]).

*ActD(t)* represents a step function of the inhibitor of transcription actinomycin D (ActD) with a value of 1 (present) or 0 (absent).

RNA polymerase II and basal transcription factors are known to be essential for transcription of genes [39], [40] whereas specific transcription factors function as enhancer or repressor. Thus we assumed in M-1 and M-2 a constitutive transcription rate for IκBα mRNA synthesis (*c_transc_*) that is independent of NFκB and is also included in the NFκB model of Hoffmann et al. [9].

Model variant M-1 was fitted to time courses of NFκB, IKKβ -P, IκBα mRNA and/or IκBα respectively gained from the following stimulation experiments:

- IL-1 (IκBα, IKKβ -P, NFκB), IL-1+UVB (IκBα, IKKβ -P, NFκB), IL1+UVB+MG132 (IκBα), UVB (IκBα), cycloheximide (CHX) (IκBα), UVB (IκBα) [15]
- IL-1+OVA (IκBα, IKKβ -P, NFκB) [18]
- ActD (IκBα), ActD+OVA (IκBα), CHX+OVA (IκBα), OVA (IκBα, IκBα mRNA), OVA+IKK knock down (IκBα)

The *in silico* knock down of IKK is realized by adding a factor named *siIKK* to the model variant and setting its value to 0.93 which reduces the initial concentration of IKK to the experimentally determined 7% of IKKβ concentration measured in untreated cells:
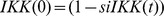



In all stimulation experiments distinct from the IKKβ knock down experiment the value of *siIKK* is set to zero.

In model variant M-2 we included the proposed IκBα complex *IκBα:Comp*, consisting of IκBα and components *Comp* distinct from NFκB:
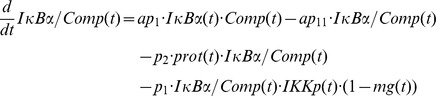


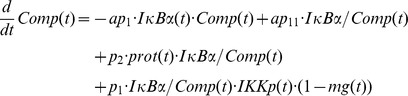



In this model variant, the activated protease *prot* additionally degrades IκBα from the IκBα complex, *IκBα:Comp*. Since this complex is part of the measured cellular IκBα concentration the overall IκBα concentration in the model, IκBα*_obs_*, becomes:
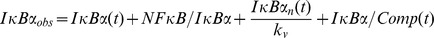



The final model, variant M-3, (Fig. 5A) was designed to investigate the possibility of IKK being part of the proposed *IκBα:Comp* complex. We therefore extended model variant M-2 by binding reactions of IκBα and IKK or IKKp and removed the component *IκBα:Comp*. In contrast to M-1 and M-2 we fitted the start concentration of IKK by adding the parameter *IKKstart*:
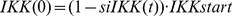



In addition we assumed that IKK as well as phosphorylated IKK is able to bind IκBα in its free and bound form.
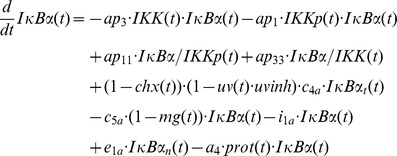


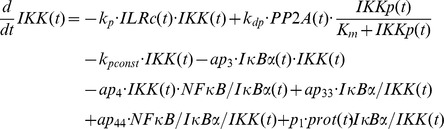


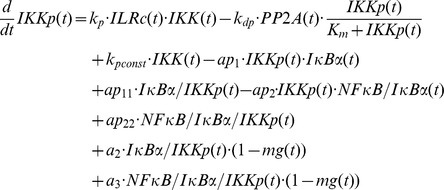


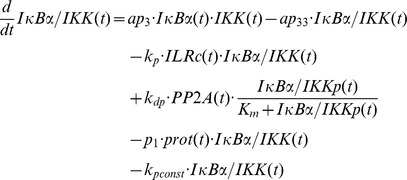


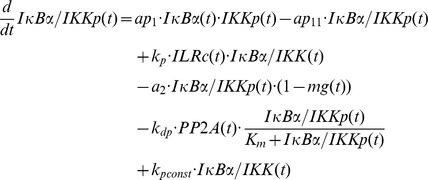


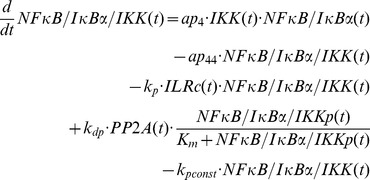


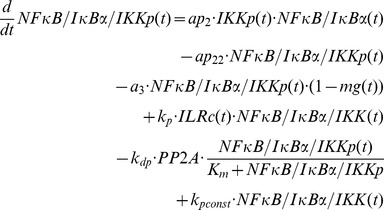



In contrast to variants M-1 and M-2 a constitutive IκBα mRNA synthesis (*c_transc_*) in M-3 is not necessarily required to reproduce all experimental data since fitting M-3 without *c_transc_* to experimental data results only in a slight decrease of the fit quality (overall χ^2^ = 100.6 compared to 98.6). Thus parameter *c_transc_* is not included in our final model M-3. This is in line with the models of Lipniacki et al. and Ashall et al. assuming that only nuclear NFκB initiates IκBα mRNA synthesis [11], [41].

Likewise, we did not integrate a constitutive degradation of IκBα in the NFκB:IκBα:IKK complex analogous to constitutive degradation of IκBα in NFκB:IκBα (*c_6a_*) since inclusion of this reaction did not improve fit quality.

The entire ODE system of each model variant is shown in Text S1, S2, S3.

For parameter estimation as well as for solving and analyzing the ordinary differential equation system we used the MATLAB (Mathworks) toolbox PottersWheel [42]. For optimization the χ^2^ value of the following objective function was minimized by using a trust region approach:
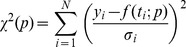



The χ^2^-value depends on the estimated parameter values. Variable y represents the i-th measured value whereas f states the simulated state value at time point i and is dependent on the parameter values p. The factor σ_i_ represents the standard deviation.

Besides the newly introduced parameters the fitted parameters of the previous model were also included in the parameter estimation with the same parameter boundaries (Table S1). Scaling parameters were included to take the lack of absolute values of the experimental data into account.

Fit sequences were applied in the estimation process: the starting value of each parameter in a sequence was thereby calculated as
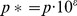



The Factor p represents the fitted parameter value of the currently best fit of the fit sequence (PottersWheel F3 routine) and the variable ε determines the strength of disturbance where ε∼N(0,n). Four subsequent runs were performed with 100 fits each using n = 4,1,0.1,0.01, respectively.

In addition we performed an identifiability analysis of the final model M-3, using the top 10% of 300 fits with random starting conditions. Many parameters are well identifiable with a low relative standard Deviation (Table S1). Furthermore for some of the less identifiable parameters, linear or non-linear correlations with other parameters exist which indicate that combinations of these parameters are identifiable (Table S1).

All models are provided as PottersWheel model datasets in the supplemental data.

## Supporting Information

Dataset S1 **PottersWheel model file of model variant M_1.**
(TXT)

Dataset S2 **PottersWheel model file of model variant M_2.**
(TXT)

Dataset S3 **PottersWheel model file of model variant M_3.**
(TXT)

Figure S1 **Overall simulation data of model variant M-1.** The 125 data points and standard deviations are depicted in blue. Simulation data is shown in red with an overall χ^2^ value of 97.
(PDF)

Figure S2 **Overall simulation data of model variant M-1 including the IKK knock down experiment.** Fitting M-1 additionally to the IKK knock down experiment increases the χ^2^ value to 129. The 127 data points and standard deviations are depicted in blue, simulation data is shown in red.
(PDF)

Figure S3 **Schematic representation of model M-2.** State variables are depicted in blue, inputs in orange and red, respectively. Reactions and variables adopted from previous models (M-1 or Witt et al. [15]) are shown in grey.
(PDF)

Figure S4 **Overall simulation data of model M-2 including the IKK knock down experiment.** The model was fitted to 127 data points and revealed an overall χ^2^ value of 119. Experimental data and standard deviation is depicted in blue. The red line represents simulation data of the best fit.
(PDF)

Figure S5 **Simulated time courses of model variant M-3 from start of integration with or without IKK knock down.** The impact of the IKK knock down on steady state concentration of free IκBα (**A**), NFκB:IκBα (**B**) and nuclear NFκB (**C**) is depicted in green. The simulated time courses without any input are shown in orange.
(PDF)

Figure S6 **Impact of a_4_, the rate constant of protease-mediated degradation of free IκBα, after IKK knock down and OVA treatment for 8 h.** Setting a_4_ to zero reveals the influence of this reaction on observed IκBα concentration (green). Reference (red) represents the simulation data of the best fit whereas experimental data and standard deviation is depicted in blue.
(PDF)

Table S1 **Description and correlation analysis of parameters of model variant M-3.** For calculation of the standard deviation (SD) and correlation analysis the best 10% of 300 fits are used.
(PDF)

Table S2 **Portions of IκBα components.**
(PDF)

Text S1 **ODE system of model variant M-1.**
(PDF)

Text S2 **ODE system of model variant M-2.**
(PDF)

Text S3 **ODE system of model variant M-3.**
(PDF)
