# Supplementary figures and images for: Identification of New IκBα Complexes by an Iterative Experimental and Mathematical Modeling Approach
Source: PLoS Comput Biol. 2014 Mar 27;10(3):e1003528. doi: 10.1371/journal.pcbi.1003528 (PMC3967930; doi:10.1371/journal.pcbi.1003528)

Supporting Figure S1

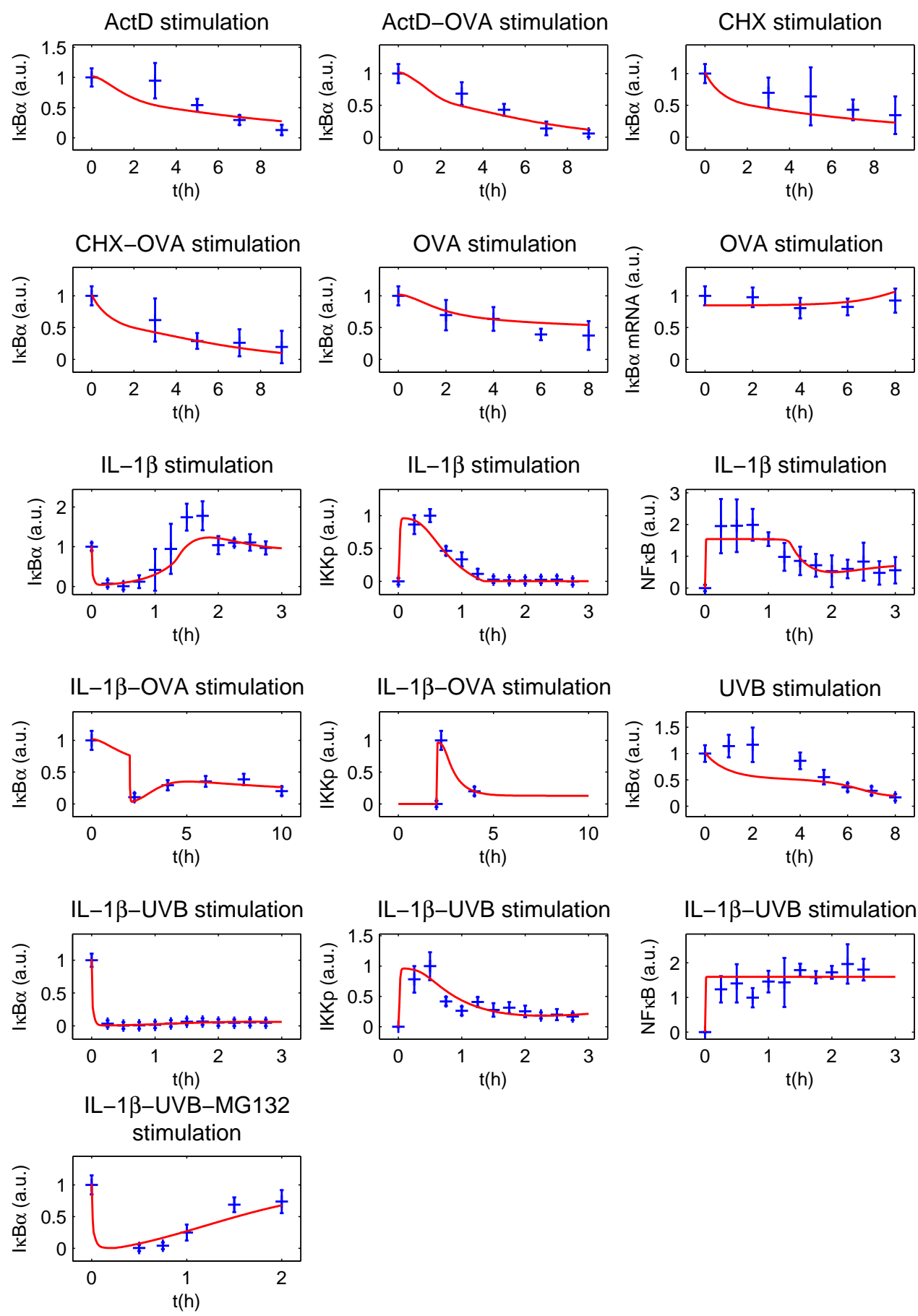

Supplement: Figure S1 — Overall simulation data of model variant M-1. The 125 data points and standard deviations are depicted in blue. Simulation data is shown in red with an overall χ2 value of 97. (PDF) [file pcbi.1003528.s004.pdf]

Supporting Figure S2

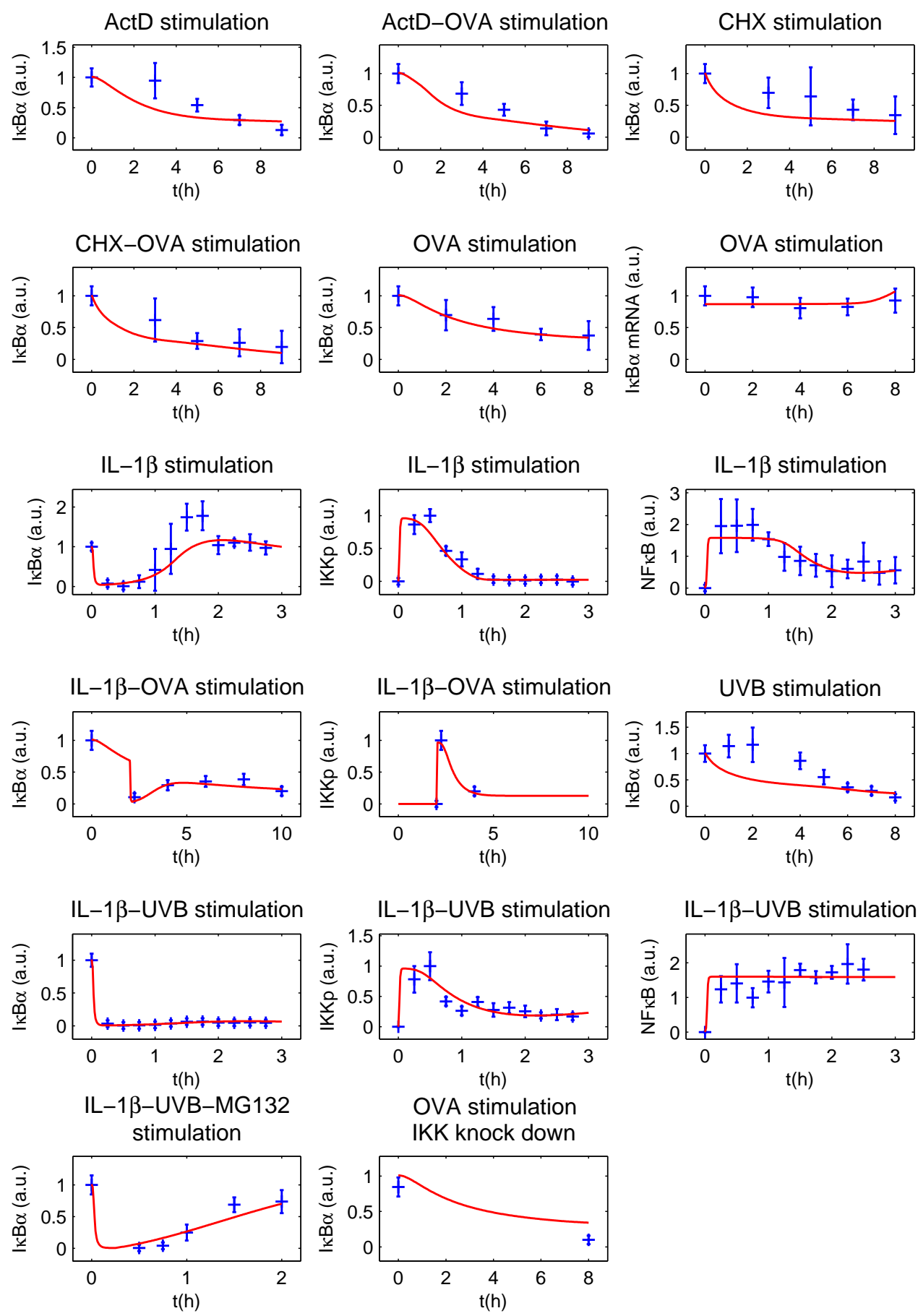

Supplement: Figure S2 — Overall simulation data of model variant M-1 including the IKK knock down experiment. Fitting M-1 additionally to the IKK knock down experiment increases the χ2 value to 129. The 127 data points and standard deviations are depicted in blue, simulation data is shown in red. (PDF) [file pcbi.1003528.s005.pdf]

Supporting Figure S3

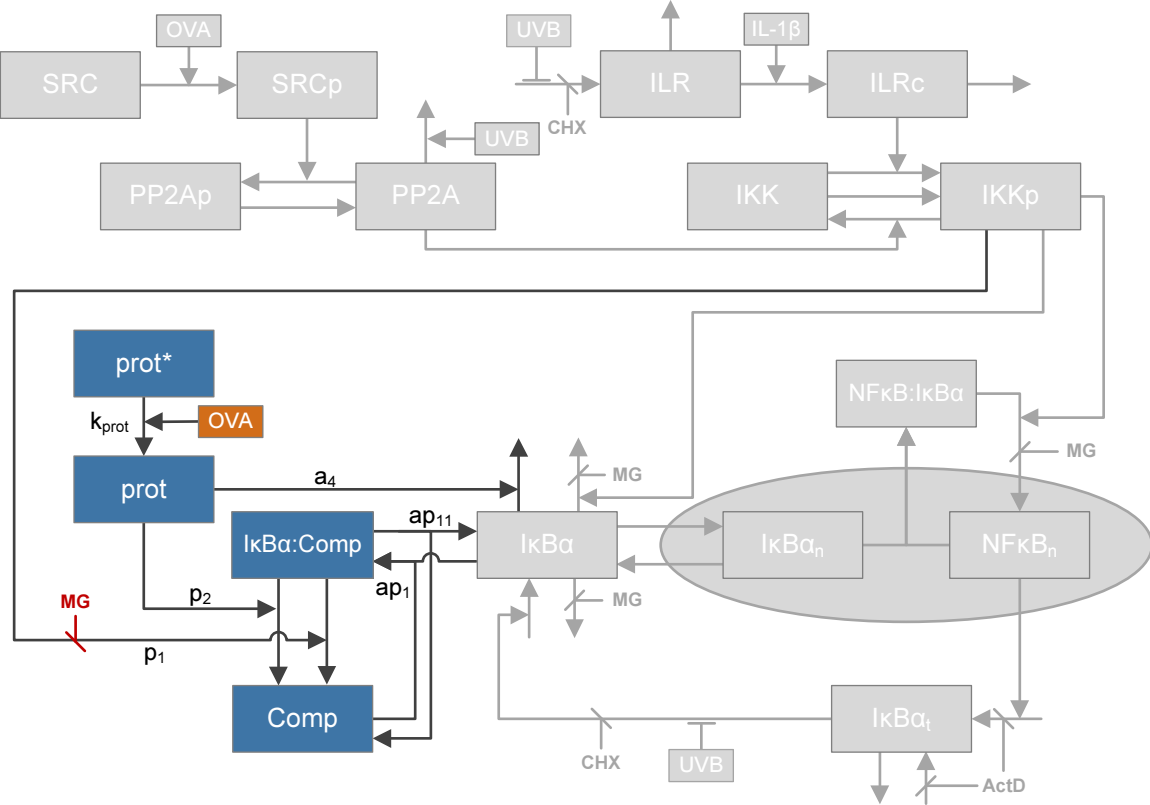

Supplement: Figure S3 — Schematic representation of model M-2. State variables are depicted in blue, inputs in orange and red, respectively. Reactions and variables adopted from previous models (M-1 or Witt et al. [15]) are shown in grey. (PDF) [file pcbi.1003528.s006.pdf]

Supporting Figure S4

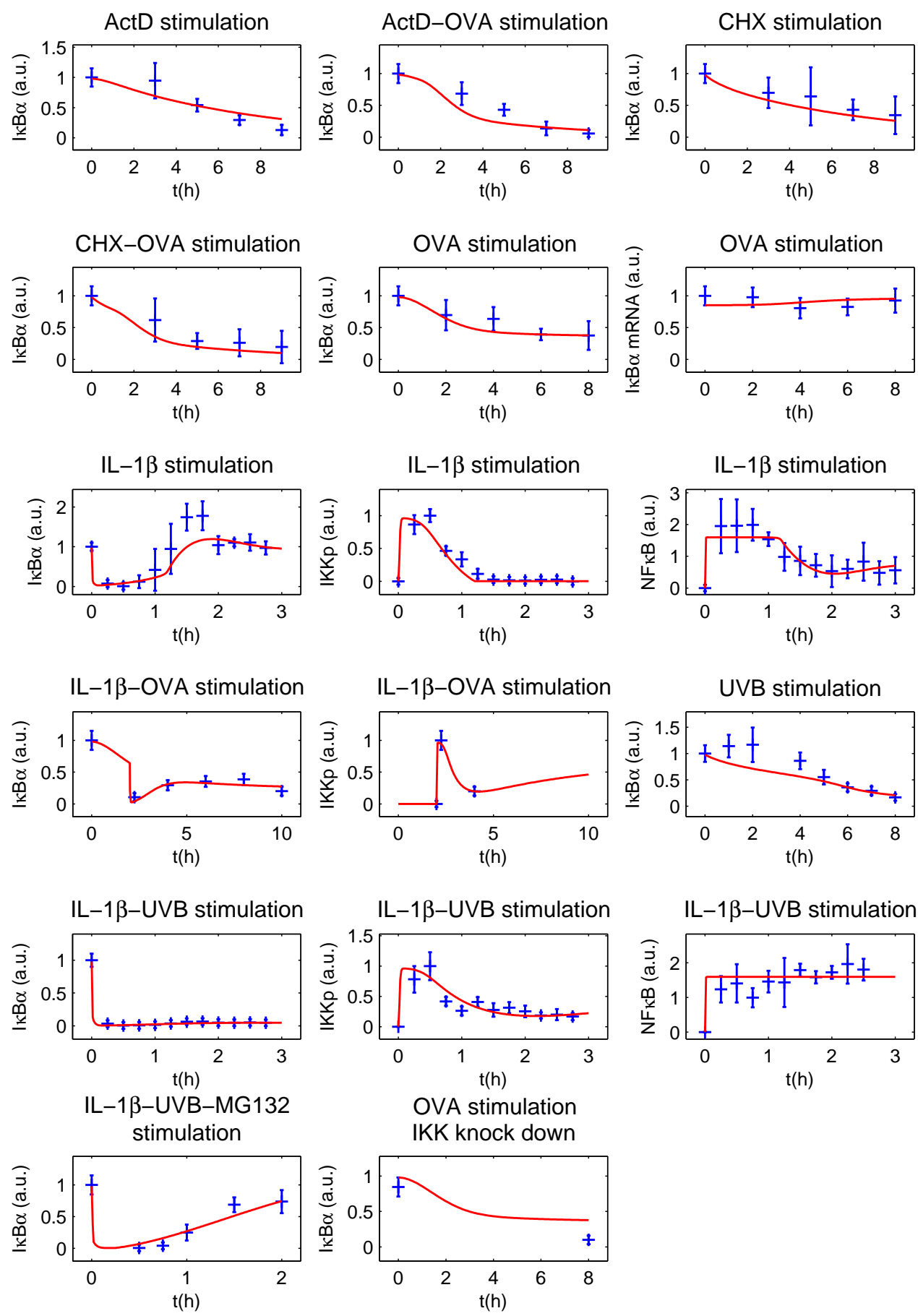

Supplement: Figure S4 — Overall simulation data of model M-2 including the IKK knock down experiment. The model was fitted to 127 data points and revealed an overall χ2 value of 119. Experimental data and standard deviation is depicted in blue. The red line represents simulation data of the best fit. (PDF) [file pcbi.1003528.s007.pdf]

Supporting Figure S5

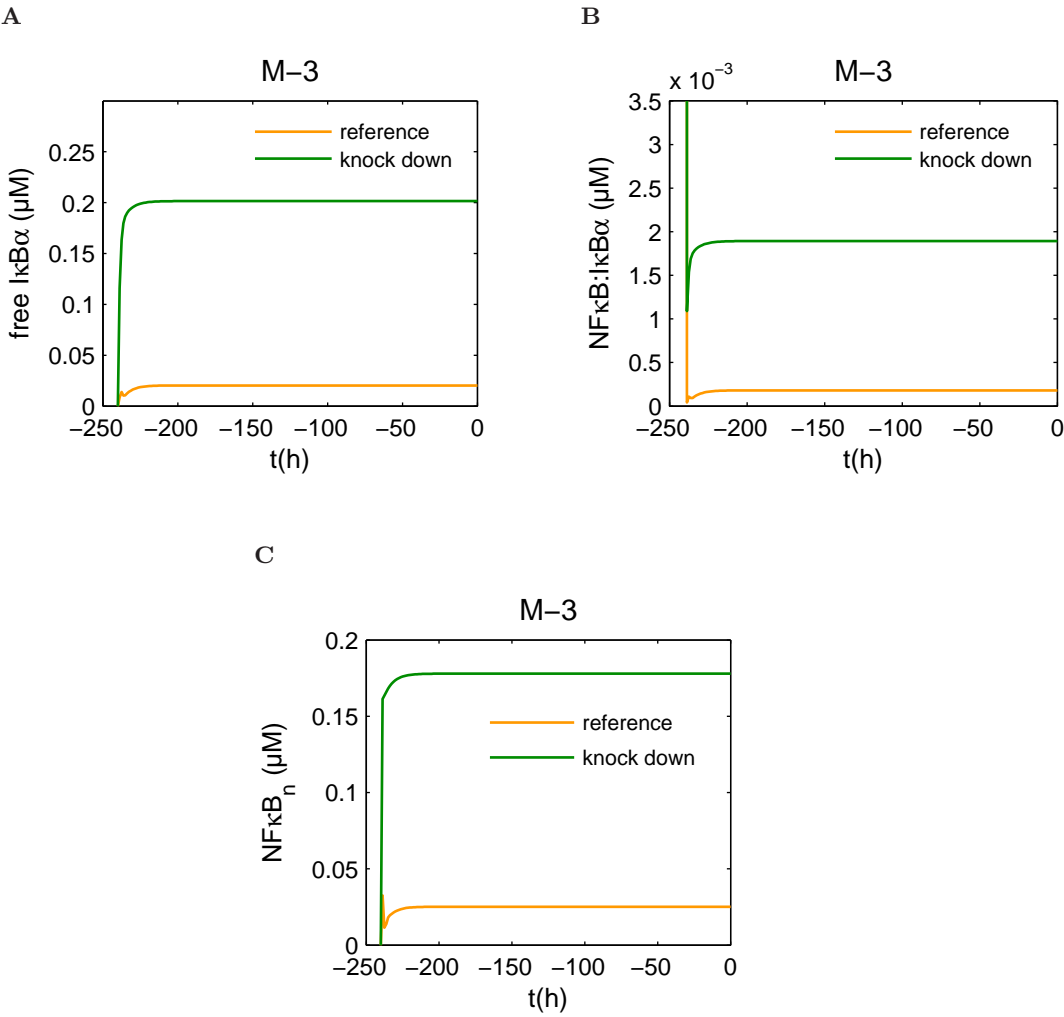

Supplement: Figure S5 — Simulated time courses of model variant M-3 from start of integration with or without IKK knock down. The impact of the IKK knock down on steady state concentration of free IκBα (A), NFκB:IκBα (B) and nuclear NFκB (C) is depicted in green. The simulated time courses without any input are shown in orange. (PDF) [file pcbi.1003528.s008.pdf]

Supporting Figure S6

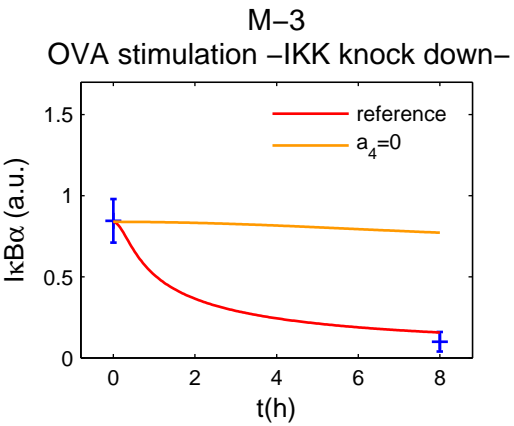

Supplement: Figure S6 — Impact of a4, the rate constant of protease-mediated degradation of free IκBα, after IKK knock down and OVA treatment for 8 h. Setting a4 to zero reveals the influence of this reaction on observed IκBα concentration (green). Reference (red) represents the simulation data of the best fit whereas experimental data and standard deviation is depicted in blue. (PDF) [file pcbi.1003528.s009.pdf]
