# Supplementary material for: Identification of New IκBα Complexes by an Iterative Experimental and Mathematical Modeling Approach
Source: PLoS Comput Biol. 2014 Mar 27;10(3):e1003528. doi: 10.1371/journal.pcbi.1003528 (PMC3967930; doi:10.1371/journal.pcbi.1003528)
Supplement: Table S1 — Description and correlation analysis of parameters of model variant M-3. For calculation of the standard deviation (SD) and correlation analysis the best 10% of 300 fits are used. (PDF) [file pcbi.1003528.s010.pdf]

Supporting Table S1

name	description	value	relative SD (%) best 10% of 300 fits	fit range	unit
a_1*	NFκB:IκBα association	1.00e+00	13	[3.00e-01,1.00e+00]	$\mu M^{-1} \cdot s^{-1}$
a_2*	degradation of IKKp bound IκBα	8.00e-01	18	[1.00e-07,8.00e-01]	s^{-1}
a_3*	degradation of IκBα in NFκB:IκBα:IKKp complex	7.83e-03	87	[1.00e-07,8.00e-01]	s^{-1}
a_4	protease mediated degradation of free IκBα	9.35e-01	37	[1.00e-09,1.00e+00]	s^{-1}
ap_1	complex formation of IκBα:IKKp	9.99e-01	16	[1.00e-04,1.00e+00]	$\mu M^{-1} \cdot s^{-1}$
ap_{11}	dissociation of IκBα:IKKp	1.54e-08	131	[1.00e-09,1.00e-04]	s^{-1}
ap_2	complex formation of NFκB:IκBα:IKKp	6.08e-03	85	[1.00e-04,1.00e+00]	$\mu M^{-1} \cdot s^{-1}$
ap_{22}	dissociation of NFκB:IκBα:IKKp	2.20e-09	162	[1.00e-09,1.00e-04]	s^{-1}
ap_3	complex formation of IκBα:IKK	3.42e-02	25	[1.00e-04,1.00e+00]	$\mu M^{-1} \cdot s^{-1}$
ap_{33}	dissociation of IκBα:IKK	4.02e-09	125	[1.00e-09,1.00e-04]	s^{-1}
ap_4	complex formation of NFκB:IκBα:IKK	1.00e+00	30	[1.00e-04,1.00e+00]	$\mu M^{-1} \cdot s^{-1}$
ap_{44}	dissociation of NFκB:IκBα:IKK	4.12e-05	29	[1.00e-09,1.00e-04]	s^{-1}
c_{1a}*	IκBα mRNA synthesis	9.20e-07	0	[1.00e-10,9.20e-07]	s^{-1}
c_{3a}*	IκBα mRNA degradation	2.66e-04	4	[2.60e-04,7.70e-04]	s^{-1}
c_{4a}*	IκBα mRNA translation	5.00e-01	0	[1.00e-07,5.00e-01]	s^{-1}
c_{5a}*	IKK-independent degradation of free IκBα	8.48e-04	15	[5.78e-04,2.00e-03]	s^{-1}
c_{6a}*	IKK-independent degradation of IκBα in NFκB:IκBα complex	7.09e-02	41	[1.00e-05,1.00e-01]	s^{-1}
e_{1a}*	IκBα nuclear export	3.35e-04	-	calculated as $i_{1a}/2$	s^{-1}
i_{1a}*	IκBα nuclear import	6.70e-04	1	[1.90e-04,6.70e-04]	s^{-1}
IKKstart	start concentration of IKK	2.61e-01	10	[2.00e-01,2.00e+00]	μM
k_a*	ILR complex formation	8.38e+00	5	[1.00e-04,1.00e+03]	$\mu M^{-1} \cdot s^{-1}$
k_{dp}*	IKK dephosphorylation	1.13e-04	8	[1.00e-07,5.00e-02]	s^{-1}
k_i*	ILR complex internalisation	3.77e-03	2	[1.00e-07,1.00e+00]	s^{-1}
k_{ilr}*	ILR turnover	9.74e-06	13	[9.20e-06,2.78e-05]	s^{-1}
K_m*	Michaelis constant for IKK dephosphorylation	1.20e-03	23	[1.00e-04,1.00e+01]	μM

**name**	**description**	**value**	**relative SD (%)** best 10% of 300 fits	**fit range**	**unit**
k_{ova}	SRC phosphorylation	7.52e-05	144	[1.00e-06,1.00e-01]	s^{-1}
k_p*	IKK phosphorylation	9.50e-02	0	[1.00e-07,9.50e-02]	s^{-1}
k_{pconst}*	constitutive IKK phosphorylation	6.35e-05	20	[1.00e-10,9.30e-03]	s^{-1}
k_{ppdp}	dephosphorylation of PP2Ap	3.89e-04	81	[1.00e-06,1.00e-02]	s^{-1}
k_{prot}	activation of protease prot	3.76e-07	43	[1.00e-09,1.00e-01]	s^{-1}
k_{src}	PP2A phosphorylation	4.89e-04	87	[1.00e-06,1.00e-01]	s^{-1}
k_{uv}*	UV-induced PP2A deactivation	2.16e-04	5	[1.00e-07,1.00e+00]	s^{-1}
k_v*	volume ratio cytoplasm/nucleus	2.90e+00	-	fix	-
NFκB:IκBα(0)	start concentration of NFκB:IκBα complex (at timepoint t=0 all NFκB:IκBα is bound to IκBα)	6.67e-02	-	fix	μM
p_1	protease mediated degradation of IKK bound IκBα	2.73e-02	38	[1.00e-09,1.00e+00]	s^{-1}
$s_{I\kappa B\alpha}$ *	IκBα scaling factor	3.77e+00	9	[1.00e-01,1.00e+02]	-
$s_{I\kappa B\alpha t}$	IκBα mRNA scaling factor	1.07e+04	10	[1.00e+03,1.00e+06]	-
s_{IKK}*	IKK scaling factor	3.63e+00	9	[1.00e-01,1.00e+02]	-
$s_{NF\kappa B1}$*	NFκB scaling factor (IL-1 stimulation)	7.69e+00	1	[1.00e-01,1.00e+02]	-
$s_{NF\kappa B2}$*	NFκB scaling factor (IL-1+UVB stimulation)	8.40e+00	1	[1.00e-01,1.00e+02]	-
uvinh*	UVB-induced translational inhibition	8.58e-01	1	[1.00e-08,1.00e+00]	-
volume*	total cell volume	2.00e+00	-	fix	pl

* Parameter adopted from Witt et al. (2012)

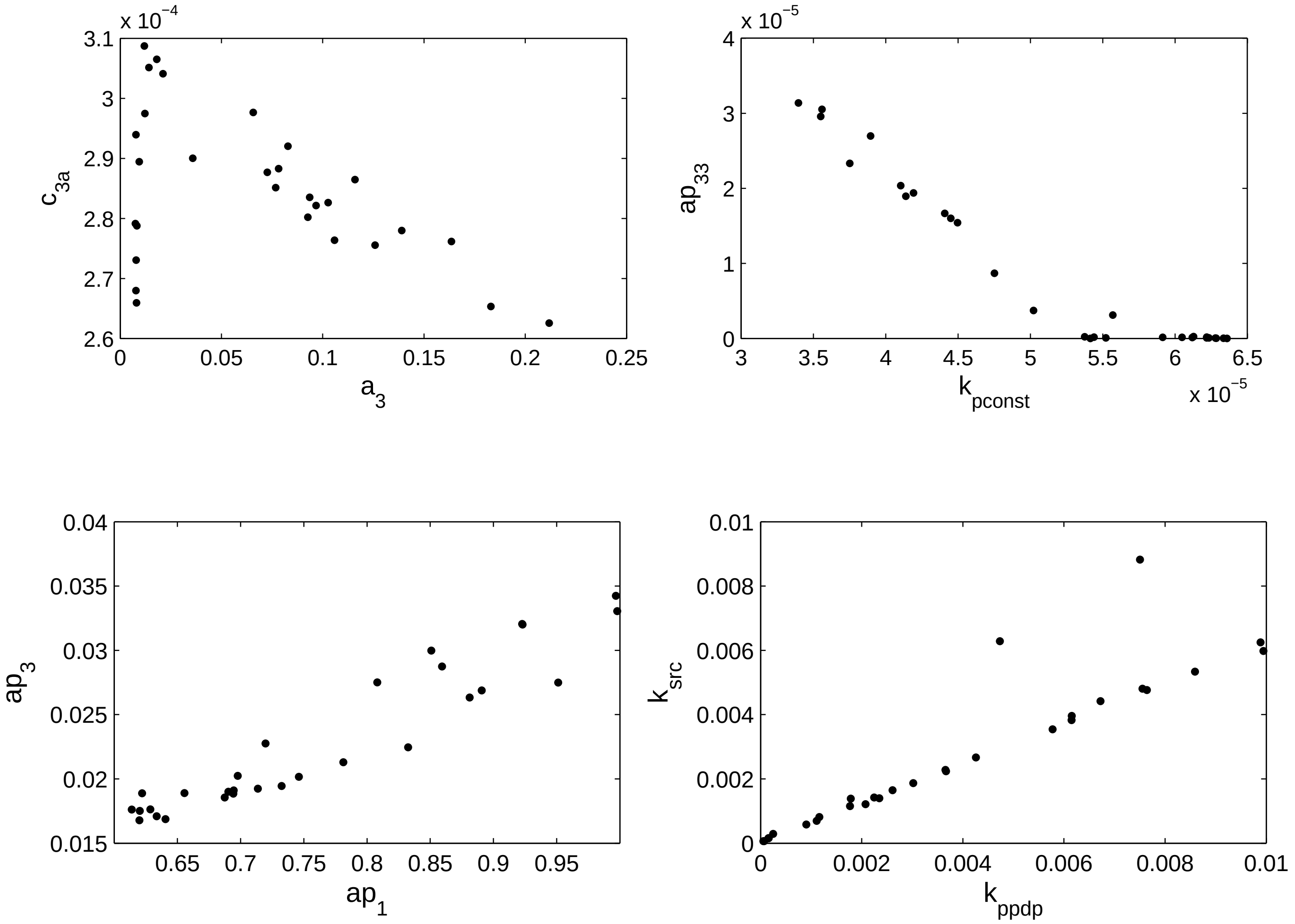
x 10^−4
3.1
3
2.9
2.8
2.7
2.6
c_3a
0
0.05
0.1
0.15
0.2
0.25
a_3
x 10^−5
4
3
2
1
0
ap_33
3
3.5
4
4.5
5
5.5
6
6.5
k_pconst
x 10^−5
0.04
0.035
0.03
0.025
0.02
0.015
ap_3
0.65
0.7
0.75
0.8
0.85
0.9
0.95
ap_1
0.01
0.008
0.006
0.004
0.002
0
k_src
0
0.002
0.004
0.006
0.008
0.01
k_ppdp
