# Supplementary material for: Identification of New IκBα Complexes by an Iterative Experimental and Mathematical Modeling Approach
Source: PLoS Comput Biol. 2014 Mar 27;10(3):e1003528. doi: 10.1371/journal.pcbi.1003528 (PMC3967930; doi:10.1371/journal.pcbi.1003528)
Supplement: Table S2 — Portions of IκBα components. (PDF) [file pcbi.1003528.s011.pdf]

Supporting Table S2

component	concentration (μM)	portion of overall IκBα (%)
**M-1:**(without fitting the model to the IKK knock down data)		
free IκBα	0.0414	30.7
NFκB:IκBα	0.0672	49.8
$I\kappa B\alpha_{n}$	0.0264	19.5
**M-2:**		
free IκBα	0.0285	12.7
NFκB:IκBα	0.0671	29.8
$I\kappa B\alpha_{n}$	0.0162	7.2
IκBα:Comp	0.1136	50.4
**M-3:**		
free IκBα	0.0203	7.60
NFκB:IκBα	1.8e-4	0.07
$I\kappa B\alpha_{n}$	5.2e-4	0.20
IκBα:IKK	0.1874	70.24
IκBα:IKKp	1.6e-5	0.01
NFκB:IκBα:IKK	0.0584	21.87
NFκB:IκBα:IKKp	3.7e-5	50.01
