# Supplementary material for: Identification of New IκBα Complexes by an Iterative Experimental and Mathematical Modeling Approach
Source: PLoS Comput Biol. 2014 Mar 27;10(3):e1003528. doi: 10.1371/journal.pcbi.1003528 (PMC3967930; doi:10.1371/journal.pcbi.1003528)
Supplement: Text S1 — ODE system of model variant M-1. (PDF) [file pcbi.1003528.s012.pdf]

Supporting Text S1

$$\frac{d}{dt}ILR(t) = -k_a \cdot il1(t) \cdot ILR(t) + (1 - uv(t) \cdot uvinh) \cdot (1 - chx(t)) \cdot k_{ilr} - ILR(t) \cdot k_{ilr}$$

$$\frac{d}{dt}ILRc(t) = k_a \cdot il1(t) \cdot ILR(t) - k_i \cdot ILRc(t)$$

$$\frac{d}{dt}IKKp(t) = k_p \cdot ILRc(t) \cdot (1 - IKKp(t)) - k_{dp} \cdot PP2A(t) \cdot \frac{IKKp}{K_m + IKKp} + k_{pconst} \cdot (1 - IKKp(t))$$

$$\frac{d}{dt}I\kappa B\alpha(t) = -a_2 \cdot (1 - mg(t)) \cdot IKKp(t) \cdot I\kappa B\alpha(t) + (1 - chx(t)) \cdot (1 - uv(t) \cdot uvinh) \cdot c_{4a} \cdot I\kappa B\alpha_t(t) - c_{5a} \cdot (1 - mg(t)) \cdot I\kappa B\alpha(t) - i_{1a} \cdot I\kappa B\alpha(t) + e_{1a} \cdot I\kappa B\alpha_n(t) - a_4 \cdot prot(t) \cdot I\kappa B\alpha(t)$$

$$\frac{d}{dt}I\kappa B\alpha_n(t) = -a_1 \cdot I\kappa B\alpha_n(t) \cdot NF\kappa B_n(t) + i_{1a} \cdot k_v \cdot I\kappa B\alpha(t) - e_{1a} \cdot k_v \cdot I\kappa B\alpha_n(t)$$

$$\frac{d}{dt}I\kappa B\alpha_t(t) = (c_{1a} \cdot NF\kappa B_n(t) + c_{transc}) \cdot (1 - ActD(t)) - c_{3a} \cdot I\kappa B\alpha_t(t)$$

$$\frac{d}{dt}NF\kappa B_n(t) = k_v \cdot a_3 \cdot IKKp(t) \cdot (1 - mg(t)) \cdot NF\kappa B|I\kappa B\alpha(t) - a_1 \cdot I\kappa B\alpha_n(t) \cdot NF\kappa B_n(t) + (1 - mg(t)) \cdot c_{6a} \cdot k_v \cdot NF\kappa B|I\kappa B\alpha(t) + k_v \cdot a_5 \cdot prot(t) \cdot NF\kappa B|I\kappa B\alpha(t)$$

$$\frac{d}{dt}NF\kappa B|I\kappa B\alpha(t) = -a_3 \cdot IKKp(t) \cdot (1 - mg(t)) \cdot NF\kappa B|I\kappa B\alpha(t) + (a_1 \cdot I\kappa B\alpha_n(t) \cdot NF\kappa B_n(t)) \cdot \frac{1}{k_v} - c_{6a} \cdot NF\kappa B|I\kappa B\alpha(t) \cdot (1 - mg(t)) - a_5 \cdot prot(t) \cdot NF\kappa B|I\kappa B\alpha(t)$$

$$\frac{d}{dt}PP2A(t) = -k_{uv} \cdot uv(t) \cdot PP2A(t) - k_{src} \cdot PP2A(t) \cdot SRCp(t) + k_{ppdp} \cdot PP2Ap(t)$$

$$\frac{d}{dt}PP2Ap(t) = k_{src} \cdot PP2A(t) \cdot SRCp(t) - k_{ppdp} \cdot PP2Ap(t)$$

$$\frac{d}{dt}SRCp(t) = k_{ova} \cdot (1 - SRCp(t)) \cdot ova(t)$$

$$\frac{d}{dt}prot(t) = k_{prot} \cdot ova(t) \cdot (1 - prot(t))$$
