# Supplementary material for: Identification of New IκBα Complexes by an Iterative Experimental and Mathematical Modeling Approach
Source: PLoS Comput Biol. 2014 Mar 27;10(3):e1003528. doi: 10.1371/journal.pcbi.1003528 (PMC3967930; doi:10.1371/journal.pcbi.1003528)
Supplement: Text S3 — ODE system of model variant M-3. (PDF) [file pcbi.1003528.s014.pdf]

Supporting Text S3

$$\frac{d}{dt}ILR(t) = -k_a \cdot il1(t) \cdot ILR(t) + (1 - uv(t) \cdot uvinh) \cdot (1 - chx(t)) \cdot k_{ilr} - ILR(t) \cdot k_{ilr}$$

$$\frac{d}{dt}ILRc(t) = k_a \cdot il1(t) \cdot ILR(t) - k_i \cdot ILRc(t)$$

$$\begin{aligned}\frac{d}{dt}IKK(t) = -k_p \cdot ILRc(t) \cdot IKK(t) + k_{dp} \cdot PP2A(t) \cdot \frac{IKKp(t)}{K_m + IKKp(t)} - \\ k_{pconst} \cdot IKK(t) - ap_3 \cdot I\kappa B\alpha(t) \cdot IKK(t) - ap_4 \cdot IKK(t) \cdot NF\kappa B|I\kappa B\alpha(t) + \\ ap_{33} \cdot I\kappa B\alpha|IKK(t) + ap_{44} \cdot NF\kappa B|I\kappa B\alpha|IKK(t) + \\ p_1 \cdot prot(t) \cdot I\kappa B\alpha|IKK(t)\end{aligned}$$

$$\begin{aligned}\frac{d}{dt}IKKp(t) = k_p \cdot ILRc(t) \cdot IKK(t) - k_{dp} \cdot PP2A(t) \cdot \frac{IKKp(t)}{K_m + IKKp(t)} + \\ k_{pconst} \cdot IKK(t) - ap_1 \cdot IKKp(t) \cdot I\kappa B\alpha(t) + ap_{11} \cdot I\kappa B\alpha|IKKp(t) - \\ ap_2 \cdot IKKp(t) \cdot NF\kappa B|I\kappa B\alpha(t) + ap_{22} \cdot NF\kappa B|I\kappa B\alpha|IKKp(t) + \\ a_2 \cdot I\kappa B\alpha|IKKp(t) \cdot (1 - mg(t)) + a_3 \cdot NF\kappa B|I\kappa B\alpha|IKKp(t) \cdot (1 - mg(t))\end{aligned}$$

$$\begin{aligned}\frac{d}{dt}I\kappa B\alpha(t) = -ap_3 \cdot IKK(t) \cdot I\kappa B\alpha(t) - ap_1 \cdot IKKp(t) \cdot I\kappa B\alpha(t) + \\ ap_{11} \cdot I\kappa B\alpha|IKKp(t) + ap_{33} \cdot I\kappa B\alpha|IKK(t) + \\ (1 - chx(t)) \cdot (1 - uv(t) \cdot uvinh) \cdot c_{4a} \cdot I\kappa B\alpha_t(t) - \\ c_{5a} \cdot (1 - mg(t)) \cdot I\kappa B\alpha(t) - i_{1a} \cdot I\kappa B\alpha(t) + \\ e_{1a} \cdot I\kappa B\alpha_n(t) - a_4 \cdot prot(t) \cdot I\kappa B\alpha(t)\end{aligned}$$

$$\frac{d}{dt}I\kappa B\alpha_n(t) = -a_1 \cdot I\kappa B\alpha_n(t) \cdot NF\kappa B_n(t) + i_{1a} \cdot k_v \cdot I\kappa B\alpha(t) - e_{1a} \cdot k_v \cdot I\kappa B\alpha_n(t)$$

$$\frac{d}{dt}I\kappa B\alpha_t(t) = c_{1a} \cdot NF\kappa B_n(t) \cdot (1 - ActD(t)) - c_{3a} \cdot I\kappa B\alpha_t(t)$$

$$\begin{aligned}\frac{d}{dt}NF\kappa B_n(t) = k_v \cdot a_3 \cdot NF\kappa B|I\kappa B\alpha|IKKp(t) \cdot (1 - mg(t)) - \\ a_1 \cdot I\kappa B\alpha_n(t) \cdot NF\kappa B_n(t) + k_v \cdot c_{6a} \cdot NF\kappa B|I\kappa B\alpha(t) \cdot (1 - mg(t))\end{aligned}$$

$$\frac{d}{dt}NF\kappa B|I\kappa B\alpha(t) = -ap_2 \cdot IKKp(t) \cdot NF\kappa B|I\kappa B\alpha(t) + ap_{22} \cdot NF\kappa B|I\kappa B\alpha|IKKp(t) - ap_4 \cdot IKK(t) \cdot NF\kappa B|I\kappa B\alpha(t) + ap_{44} \cdot NF\kappa B|I\kappa B\alpha|IKK(t) + (a_1 \cdot I\kappa B\alpha_n(t) \cdot NF\kappa B_n(t)) \cdot \frac{1}{k_v} - c_{6a} \cdot NF\kappa B|I\kappa B\alpha(t) \cdot (1 - mg(t))$$

$$\frac{d}{dt}PP2A(t) = -k_{uv} \cdot uv(t) \cdot PP2A(t) - k_{src} \cdot PP2A(t) \cdot SRCp(t) + k_{ppdp} \cdot PP2Ap(t)$$

$$\frac{d}{dt}PP2Ap(t) = k_{src} \cdot PP2A(t) \cdot SRCp(t) - k_{ppdp} \cdot PP2Ap(t)$$

$$\frac{d}{dt}SRCp(t) = k_{ova} \cdot (1 - SRCp(t)) \cdot ova(t)$$

$$\frac{d}{dt}prot(t) = ova(t) \cdot k_{prot} \cdot (1 - prot(t))$$

$$\frac{d}{dt}I\kappa B\alpha|IKK(t) = ap_3 \cdot I\kappa B\alpha(t) \cdot IKK(t) - ap_{33} \cdot I\kappa B\alpha|IKK(t) - k_p \cdot ILRc(t) \cdot I\kappa B\alpha|IKK(t) + k_{dp} \cdot PP2A(t) \cdot \frac{I\kappa B\alpha|IKKp(t)}{K_m + I\kappa B\alpha|IKKp(t)} - p_1 \cdot prot(t) \cdot I\kappa B\alpha|IKK(t) - k_{pconst} \cdot I\kappa B\alpha|IKK(t)$$

$$\frac{d}{dt}I\kappa B\alpha|IKKp(t) = ap_1 \cdot I\kappa B\alpha(t) \cdot IKKp(t) - ap_{11} \cdot I\kappa B\alpha|IKKp(t) + k_p \cdot ILRc(t) \cdot I\kappa B\alpha|IKK(t) - a_2 \cdot I\kappa B\alpha|IKKp(t) \cdot (1 - mg(t)) - k_{dp} \cdot PP2A(t) \cdot \frac{I\kappa B\alpha|IKKp(t)}{K_m + I\kappa B\alpha|IKKp(t)} + k_{pconst} \cdot I\kappa B\alpha|IKK(t)$$

$$\frac{d}{dt}NF\kappa B|I\kappa B\alpha|IKK(t) = ap_4 \cdot IKK(t) \cdot NF\kappa B|I\kappa B\alpha(t) - ap_{44} \cdot NF\kappa B|I\kappa B\alpha|IKK(t) - k_p \cdot ILRc(t) \cdot NF\kappa B|I\kappa B\alpha|IKK(t) + k_{dp} \cdot PP2A(t) \cdot \frac{NF\kappa B|I\kappa B\alpha|IKKp(t)}{K_m + NF\kappa B|I\kappa B\alpha|IKKp(t)} - k_{pconst} \cdot NF\kappa B|I\kappa B\alpha|IKK(t)$$

$$
\begin{aligned}
\frac{d}{dt}NF\kappa B|I\kappa B\alpha|IKKp(t) = ap_2 \cdot IKKp(t) \cdot NF\kappa B|I\kappa B\alpha(t) - \\
ap_{22} \cdot NF\kappa B|I\kappa B\alpha|IKKp(t) - a_3 \cdot NF\kappa B|I\kappa B\alpha|IKKp(t) \cdot (1 - mg(t)) + \\
k_p \cdot ILRc(t) \cdot NF\kappa B|I\kappa B\alpha|IKK(t) - \\
k_{dp} \cdot PP2A \cdot \frac{NF\kappa B|I\kappa B\alpha|IKKp(t)}{K_m + NF\kappa B|I\kappa B\alpha|IKKp} + \\
k_{pconst} \cdot NF\kappa B|I\kappa B\alpha|IKK(t)
\end{aligned}
$$
